# Effective tax rates of multinational corporations: Country-level estimates

**DOI:** 10.1371/journal.pone.0293552

**Published:** 2023-11-29

**Authors:** Javier Garcia-Bernardo, Petr Janský, Thomas Tørsløv

**Affiliations:** 1 Department of Methodology & Statistics, Utrecht University, Utrecht, The Netherlands; 2 Centre for Complex Systems Studies, Utrecht University, Utrecht, The Netherlands; 3 Institute of Economic Studies, Faculty of Social Sciences, Charles University, Prague, Czechia; 4 Danmarks Nationalbank, Copenhagen, Denmark; Budapest Business School University of Applied Science: Budapesti Gazdasagi Egyetem, HUNGARY

## Abstract

Effective tax rates (ETRs) estimated from the income statement data of multinational corporations (MNCs) are useful for comparing MNCs’ corporate income taxation across countries. In this paper, we propose a new methodological approach to estimate ETRs as reliably and for as many countries as possible using Orbis’ unconsolidated data for the 2011–2015 period. We focus on countries with at least 50 available companies, which results in a sample of 47, mostly European, countries. We estimate the ETR of a country as the ratio of corporate income tax to gross income for all affiliates of MNCs in that country, weighted by gross income. We propose four ETR estimations, including lower and upper bounds, which differ by gross income calculation. We find that ETRs substantially differ from statutory tax rates for some countries. For example, we show that despite similar statutory rates of 28% and 29%, MNCs in Luxembourg paid as little as 1–8% of gross income in taxes, while those in Norway paid as much as 46–67%. Despite being the best available, existing data is still imperfect. We therefore call for better data in the form of MNCs’ unconsolidated, public country-by-country reporting data.

## 1. Introduction

Tax avoidance by multinational corporations (MNCs) contributes to inequalities both between and within countries. When MNCs shift profits to tax havens, other countries receive less profit and lower tax revenues [1, 2]. If MNCs avoid taxes in a given country, the tax burden can be transferred to other taxpayers [3]. This tax avoidance has been addressed by the two-pillar proposal coordinated by the [4] and agreed as a common approach by more than 100 countries in 2021. Pillar 1 [5]—similarly to other proposals in the European Union [6–8] or globally [9]—would redistribute some MNCs’ profits according to economic activity. Responding to tax avoidance, Pillar 2 takes a more direct approach to ensuring MNCs pay some taxes [5]. It requires MNCs to pay a minimum effective tax rate (ETR) of 15% in each country that implements it. In December 2022, the European Union agreed that Pillar 2 will be mandatory for its members from 2024. As a result, the taxes paid by MNCs are likely to increase, though it is not clear how much MNCs pay in taxes now.

Existing studies have attempted to establish how much MNCs pay in taxes primarily by relying on one of two conceptually different approaches to ETRs. One uses a model of hypothetical companies developed on the basis of existing legislation [10, 11], which results in forward-looking ETRs. On the one hand, these forward-looking ETRs have been estimated extensively for EU member states [12], G20 countries [13], and OECD members [14], and they provide important policy insights and useful data for research purposes. On the other hand, however, they seldom focus on MNCs, and their estimates are by definition based on hypothetical modelling rather than on the observed behaviour of companies. Consequently, they are unsuitable for determining the *actual* tax rate that MNCs pay in a country.

The second approach uses data available for existing companies to develop backward-looking ETRs (or simply ETRs in this paper), which constitute our focus. In addition to Orbis, which we use in our paper, US-focused sources such as Form 5471 by [15] or Compustat by [16, 17] or [18], are among other relevant data sources (reviewed more comprehensively by [19]. We believe that the potential of backward-looking ETRs has gone untapped thus far and that their use in research has been limited by their availability. At present, no established source of backward-looking MNC ETRs is readily available or widely used. Indeed, to our knowledge, no reliable and continuously-updated databases of ETRs estimated using company data are currently available. The lack of these ETRs may be explained by both data- and methodology-related obstacles. For example, even the best available datasets suffer from poor accuracy and limited coverage. Even if the data were perfect, the very existence of multiple methodological approaches designed to estimate ETRs constitutes an issue in itself. Thus, there is naturally less consensus on how to estimate ETRs than on how to determine the statutory tax rate. In this paper, we overcome these challenges and fill the gap by developing a new methodological approach to reliably estimate ETRs for as many countries as possible.

In particular, we identify how much MNCs pay in corporate income taxes in various countries by estimating ETRs based on MNCs’ income statement data. The ETRs of these MNCs show how much they pay in corporate income tax. In addition, we compare estimated ETRs across countries and compare them with statutory corporate income tax rates. To provide as reliable ETR estimates as possible, we use unconsolidated data on MNCs between 2011 and 2015 provided by Orbis, which has the best data coverage for Europe and a good one—with at least 50 companies—for 47 countries worldwide. While we need to consider some limitations of Orbis, such as the lack of detailed financial profit data discussed below, it has the best European and worldwide coverage and its data concepts are unified across countries. Additionally, in the absence of country-specific administrative tax data, Orbis is also likely the first choice of both researchers and policymakers when it comes to measuring ETRs or evaluating the impact of reforms, such as the global minimum tax.

We find that the amount of taxes accrued by MNCs varies considerably from country to country. We also highlight substantial differences between ETRs faced by MNCs and statutory corporate income tax rates. As an extreme example, we show that, despite similar statutory rates of 28% and 29%, MNCs in Luxembourg paid as little as 1–8% of gross income in taxes, while those in Norway paid as much as 45–66% (driven mostly by the oil sector, as we discuss below). Our novelty is not necessarily in highlighting which countries, such as Luxembourg, offer the lowest effective taxation—as there is some research suggesting this using both narrow [20, 21] and broader measures of corporate taxation [22, 23]. Rather, we show how low these ETRs are. Furthermore, despite our focus on estimating backward-looking ETRs, we compare them with one set of leading estimates of forward-looking ETRs prepared by the OECD [24]. These ETRs also relate to Pillar 2 and our estimates indicate which countries might be affected by the minimum tax of 15%.

Our results are the first to reveal the actual extent of the differences between ETRs and statutory tax rates for many countries. At the same time, existing evidence explains why ETRs of MNCs are expected to be lower than statutory rates or ETRs of domestic firms in most cases, given the ample range of tax credits and incentives such as tax holidays [21, 25–28]. Our results also suggest that some EU countries do not tax MNCs much and that Luxembourg, for example, cannot lower its effective taxation much further since its ETRs are already close to zero. This is consistent with a race to the bottom with respect to ETRs and contributes to the existing evidence on this phenomenon in the European Union and the rest of the world [29–33].

Our main contribution is a new methodology that estimates country-level backward-looking ETRs using the most extensive unconsolidated data of companies (Orbis) while carefully reflecting its limitations. As far as we know, this is the first paper explicitly dedicated to estimating ETRs from Orbis data for many countries, which enables us to estimate as thoroughly as possible. This contrasts with most existing literature, which estimates ETRs using Orbis as one of the steps in more complex research that often lacks a thorough estimation methodology such as the one we provide. Similarly to [34–36] we use unconsolidated Orbis data to estimate ETR as the ratio of corporate income tax to gross income for all affiliates of MNCs in a given country, weighted by gross income. In contrast to any previous research, we propose four ETR estimations, including lower and upper bounds, that differ by gross income calculation. We outline the explanations for these four estimations and why researchers using unconsolidated Orbis data to estimate ETRs should reflect them, regardless of which estimation they choose. While our main goal is to provide a new methodology to estimate ETRs using Orbis data that can be used by other researchers—or against which they can benchmark their preferred methodology—we also apply our methodology to provide a new set of estimates. These shed new light on corporate taxation across many European and other countries and are relevant for tax reforms. When considering the effects of any reform of MNC taxation, we first need to establish the status quo, starting with how much tax MNCs actually pay.

The remainder of this paper is structured as follows. In Section 2 Data, we discuss the characteristics of Orbis data. In Section 3 Methodology, we outline our straightforward approach to estimating ETRs using unconsolidated data. In Section 4 Results, we present the results and discuss the differences between estimated ETRs and statutory tax rates. In Section 5 Conclusion, we briefly examine the implications of our results for policy and future research. The S1 Appendix includes additional figures and the results of our robustness checks.

## 2. Data

We use the Orbis database to estimate ETRs across countries and over time. Orbis is a commercial product of the Bureau van Dijk company and one of the best company-level data sources available. Its coverage of companies since the mid-2000s is quite comprehensive, with information for some companies available since the 1980s. Orbis has been widely used in economic literature and contains both consolidated and unconsolidated data, used for example earlier by [37] and recently by [38] to study profit shifting. Unconsolidated data may be also used to estimate ETRs for individual affiliates and thus to estimate how much tax MNCs pay in individual countries, which is why we use them in this paper. On the other hand, while consolidated data may be used to estimate ETRs at company group level, they cannot be disaggregated by country and are therefore not used in this paper. Orbis data is financial, rather than tax, reporting and therefore the literature on book-tax conformity for MNCs is relevant [39, 40]. For example, [41] hints at the scale of this difference by comparing tax returns data with accounting data for the United Kingdom: she finds that using accounting data overestimates the ratio of taxable profits to total assets by foreign multinational affiliates.

We use the latest Orbis version available to us at the time of research. We utilize the fullest Orbis data as accessed in December 2017; in this case, the latest year with available data is 2016 (which had poor coverage) and the best five-year period is 2011 to 2015. Our study of ETRs is limited to this five-year-long period to allow tax credits awarded by losses to offset tax liabilities. Key variables associated with unconsolidated data used in Orbis are profit (“P/L before Tax” in Orbis), tax (“Taxation”), operating profit (“Operating P/L [= EBIT] (equal to P/L before Tax–Financial profit)”), financial revenue (“Financial revenue”) and financial expenses (“Financial expenses”).

We now clarify the process of data selection using the Orbis company database (Table 1). We start with a sample of 15,684,360 companies and 60,400,740 observations between 2011 and 2015, of which 1,682,851 companies and 5,915,908 observations are affiliates of MNCs. We determine MNCs using the ID of the global ultimate owner (GUO); we consider GUOs with affiliates in more than one country as MNCs. We further reduce the sample of all MNCs. First, due to their specific behaviour and distribution across countries, we exclude all financial companies by keeping the affiliates of Orbis type “C” (corporate) companies. Second, by including only private limited companies and public limited companies (removing mainly partnerships) we arrive at a sample which is more comparable across countries, and which allows us to establish the tax payment of the ETR of an average MNC’ affiliate.

**Table 1:** Orbis data sample: Non-financial MNC affiliates with limited liability.

Restrictions in data	Number of affiliates	% of affiliates	Number of observations	% of observations
MNCs 2011–2015	1,682,851	(100.0%)	5,915,908	(100.0%)
1. Drop financial:	794,914	(47.2%)	2,617,019	(44.2%)
2. Drop non-limited:	533,046	(31.7%)	1,818,415	(30.7%)
3. Drop company if negative profits in 2010:	489,199	(29.1%)	1,654,808	(30.0%)
4. Drop last year if negative profits:	272,321	(16.2%)	905,255	(15.3%)
5. Drop negative tax or profit or ETR over 1, aggregate by affiliate:	198,527	(11.8%)		

Notes: Authors on the basis of Orbis, 2011–2015. Several years are available for each affiliate.

The overriding objective of the remaining restrictions is to minimise the effect that losses (negative profits) have on the estimation of ETRs. To clarify, the goal is to calculate the ETR paid by MNCs in normal business circumstances, i.e., those mostly profitable, not necessarily the average ETR of all affiliates (including many affected by losses). So, third, since the goal of the paper is to calculate the ETR paid by normal MNC, we want to remove the effect of carry-forward loses and outliers. To reduce the effects of losses from earlier years carried forward (which often leads to positive taxation, i.e., taxes received rather than paid, in subsequent years), we drop company if it had negative profits in 2010. Fourth, we drop observations with negative profits if the observation occurs in the last year of the sample (we carry out a robustness check which shows that the results are broadly similar with and without this adjustment, see Table A1 in the S1 Appendix).

Fifth and finally, we also delete all companies with negative profits or taxes for the entire period, and companies with ETRs over the value of one. We drop the final year if it has negative profits, since it ensures that there is enough time to account for carry-over losses and that the denominator of the ETR (sum of profits) is consistent, ensuring that the ETR is not distorted (reduced) by losses. The advantage of these adjustments is that negative taxes or taxes higher than profits do not make good economic sense, perhaps except for a merger of two companies, where some tax arrangement can lead to negative taxation, or other forms of restructuring which we are not able to capture properly using existing data. The cases of errors in the Orbis data are another possibility. The disadvantages are that deleting observations with negative taxes might inflate ETRs and that deleting observations with ETRs above one might deflate ETRs. These adjustments result into a final sample of 198,527 MNCs’ affiliates.

In addition, we run several data-related robustness tests: In Table A1 in the S1 Appendix we do not exclude observations for companies with negative profits in the previous year. In Table A2 in the S1 Appendix we use a balanced panel data set for all four ETR versions. In Table A3 in the S1 Appendix we apply both these conditions simultaneously. In Table A4 in the S1 Appendix we keep only affiliates with 3 or more observations between 2011 and 2015.

Overall, our results are based on the following final sample: the data sample includes 47 countries with available data for a minimum of 50 companies (out of a total of up to 93 countries with at least one company in Orbis; we also present results for all these countries in tables below for the sake of completeness and as a check on the expertly selected cut-off of a minimum of 50 companies). While the sample includes all EU member states, it unfortunately does not include the US due to its poor coverage in the Orbis database (only one company included).

We use Orbis since it constitutes the most suitable source for a cross-country analysis of MNCs’ unconsolidated data. We selected it based on a detailed study of Orbis and alternative data sources. Although Orbis is one of the best available data sources, it does suffer from a number of shortcomings. We discuss in Section 3 Methodology below how we deal with one such limitation associated with the low level of detail relevant to financial profits data available in Orbis (in contrast to Orbis, some country-specific data sources such as Dafne for Germany provide more detailed information and overcome similar limitations). Additional limitations of Orbis are discussed by [42–50]. For example, its coverage of individual companies is not universal and differs from country to country—it is, for example, biased against tax havens and developing countries. Even for included companies, the amount of available information differs and is frequently limited. [21] show that only a weighted average of 17% of global (consolidated) profits is included in the unconsolidated accounts. Furthermore, the Orbis database is unable to sufficiently account for the specific characteristics of various tax systems—e.g. deferred taxes are of relatively low quality, although we do address this issue in part by using a five-year period. Similarly, the Orbis database does not account for specifics of individual corporate income tax systems (e.g. up to six sevenths of the corporate tax paid in Malta can be claimed as refunds to shareholders). In view of these Orbis limitations, any results based on this database, including ours, are limited; as such, they should not be used as the only evidence base for policymakers’ decisions.

In addition to Orbis company data, we use several other data sources. In Section 4 Results we compare ETRs with headline statutory corporate income tax rates, which generally constitute the most frequently applied or the highest applicable statutory rates. These statutory rates are sourced primarily from a corporate income tax database published by [51], supplemented by additional sources when needed [52]. We also compare our backward-looking ETRs with forward-looking ETRs from OECD available for 2017 [24] in three interest and inflation rate scenarios: low, high and country-specific.

## 3. Methodology

In this paper we develop a new methodological approach to estimate four versions of ETRs of MNCs’ affiliates. All four versions are based on the same logic: for each MNC affiliate, we divide its corporate income tax by its gross income to arrive at its ETR. We then calculate a gross income-weighted mean of the ETRs of all affiliates located in one country. In this way we arrive at a country-level ETR for all affiliates of all MNCs in one country. In effect, this is a gross income-weighted mean of ETRs of MNCs’ affiliates located in one country.

ETR estimation is complicated by the fact that information on gross income is not unambiguously available in the data. For ETR estimation purposes, gross income would ideally comprise corporate income taxes, operating profits, and only some financial profits. Such financial profits would ideally include interest (as well as other income such as royalties) but not equity income. This is because while interest is generally taxable–and should thus be included in gross income, equity income is not–and should therefore be excluded from gross income. If available data distinguished between interest and equity income, we could estimate “true ETR” values by excluding equity income from its denominator. No such distinction is available for most companies. Orbis provides only three relevant indicators with good availability: financial revenue, financial expenses, and financial profit (financial profit is financial revenue minus financial expenses). Each of the three variables lumps together both interest and equity income (a separate variable does exist for interest paid, but we do not use it since there is no variable for interest received). To address this inherent limitation of Orbis data, we calculate four separate versions of ETR estimates: two point estimates as well as lower and upper bound estimates.

While each of our two point estimates makes good economic sense, neither likely produces a true ETR value owing to its construction. To calculate ETR1 we include both financial revenue and financial expenses in the denominator, thereby also including equity income which should ideally be excluded since it is likely not taxable. This is the preferred version since many affiliates have substantial interest expenses, but no substantial equity income—see the OECD/G20 Inclusive Framework’s Economic Analysis and Impact Assessment [53] By contrast, to calculate ETR2 we exclude both financial revenue and financial expenses from the denominator of, thus excluding not only equity income, but also interest income, which is likely taxable and should be included. The two main ETR estimates are calculated using unconsolidated company data for each country *i* and year *t* as follows:

$${ETR1}_{it}=\frac{\sum {Corporate\;income\;tax}_{it}}{\sum {\left(Operating\;profit+Financial\;profit\right)}_{it}}$$

$${ETR2}_{it}=\frac{\sum {Corporate\;income\;tax}_{it}}{\sum {\left(Operating\;profit\right)}_{it}}$$

where the sum of corporate income taxes constitutes unconsolidated taxes accounted for in the income statements of MNC affiliates located in country *i* and the sum of gross incomes constitutes a sum of these taxes and the remaining unconsolidated gross income accounted for in the income statements of MNC affiliates located in country *i*. Gross income in ETR1 includes corporate income taxes, operating profit along with financial revenue and financial expenses, i.e. the sum of interest income (taxable in country *i*) and equity income (generally not taxable in country *i*). On the other hand, gross income in ETR2 only includes operating profit in addition to the taxes.

Since both point estimates constitute logical approaches to the presented data challenge, we present both sets of results as the two best estimates of true ETR values. Since the interest and equity income variables may attain both positive and negative values, ETR1 may be either lower or higher than ETR2 (indeed, ETR1 is lower than ETR2 for 29 of the 47 countries, see Table 2 below). As a result, neither ETR1 nor ETR2 can function as either a lower or an upper bound for the true ETR value and additional estimates are thus needed.

**Table 2:** Effective tax rates.

Country	EU	ISO2	CIT (%)	Mean (%)				Median (%)				Number of companies			
				ETR1	ETR2	ETR3	ETR4	ETR1	ETR2	ETR3	ETR4	ETR1	ETR2	ETR3	ETR4
**Albania** *	Other Europe	AL	12	14.4				14.4				1			
**Algeria** *		DZ	25	25	19.3	17.6	13.1	23.2	21.6	19.7	24.9	33	32	33	30
**Argentina** *		AR	35	38.1	38	31.5	48.4	35.8	36.6	33.4	39.1	31	22	26	17
**Armenia** *		AM	20	22				22				1			
**Australia**		AU	30	30.6	28.4	27	32.8	29.8	28.4	26.8	30.8	1349	1339	1224	1054
**Austria**	EU27	AT	25	12.7	17.1	11	18.3	21.2	22.4	17.8	25	1771	1659	1810	1499
**Barbados** *		BB	25	1.8		1.8		1.8		1.8		1		1	
**Belgium**	EU27	BE	34	13.3	21.6	6.8	29.2	28.7	29.1	22.3	34.6	5670	5333	5872	4689
**Bermuda** *		BM	0	18.6	36.8	16.8	46.5	18.6	36.8	16.8	46.5	1	1	1	1
**Bolivia** *		BO	25	37.5				37.5				1			
**Bosnia and Herzegovina**	Other Europe	BA	10	7	7.1	6.4	7.7	9.5	9.3	8.7	10.1	274	266	281	252
**Brazil**		BR	34	24.1	21.7	17.8	29.5	28.9	22.9	19.1	33.4	376	352	380	310
**Bulgaria**	EU27	BG	10	9.6	8.4	5.6	10.9	10.1	9.5	8.8	10.6	790	767	817	717
**Burkina Faso** *		BF	16	32.9	30	29.7	33.3	32.9	30	29.7	33.3	1	1	1	1
**Cambodia** *		KH	20	4.6				4.6				1			
**Canada** *		CA	26.6	21.8				21.8				1			
**Cape Verde** *		CV	25	24.5	22.1	21.6	25.2	24.5	22.1	21.6	25.2	1	1	1	1
**Chile** *		CL	20.5	19.3	19.8	14.3	23.9	18.6	17	13.6	22.2	22	19	21	18
**China**		CN	25	19	19.6	17.3	21.9	21.2	20.2	19.5	22.6	3884	3858	3910	3792
**Colombia**		CO	28.2	30.1	29.9	22.1	38.2	33.2	33.1	26.9	37.5	597	600	207	174
**Costa Rica** *		CR	30	37.5				37.5				1			
**Cote d’Ivoire** *		CI		25.8	25	25	25.8	25.8	25	25	25.8	1	1	1	1
**Croatia**	EU27	HR	20	14	13.5	10.3	18.2	20.3	19	16	22	980	980	1054	841
**Cyprus** *	EU27	CY	11.5	6.2	6.7	5.5	7.5	12	9.4	8.3	12.4	43	43	39	38
**Czechia**	EU27	CZ	19	13.8	17	10.7	20.1	19.4	18.3	15.4	22	4285	4296	4488	3678
**Denmark**	EU27	DK	24.3	15.8	28.6	12.9	33.4	24.3	24.2	19.4	26.1	4017	3341	4131	3012
**Dominica** *		DM		28.2	22.4		28.2	28.2	22.4		28.2	1	1		1
**Ecuador** *		EC	22.6	23.3	22.6	21.9	25.2	25.1	21.9	20	25.6	8	8	8	8
**Egypt** *		EG	23.5	58.1	58.1	57.6	58.4	58.1	58.1	57.6	58.4	1	1	1	1
**El Salvador** *		SV	30	40	28.3	22.3	43.1	39.8	28.7	23.5	43.1	2	2	2	1
**Estonia**	EU27	EE	20.8	14.1	14.6			15.1	15.3			551	544		
**Finland**	EU27	FI	23	12.3	16.2	9.8	17.3	21.9	21.9	19.9	23.3	2393	2305	2402	2144
**France**	EU27	FR	33.3	16.2	31	12.1	34.2	27.7	28.3	24.4	30.9	12764	11867	13104	11081
**Gabon** *		GA		32.4	28.3	27.6	32.6	32.4	28.3	27.6	32.6	1	1	1	1
**Germany**	EU27	DE	29.5	19.6	23.7	14.8	27.8	27.3	26.2	21.9	30.1	7728	7361	7908	6592
**Greece**	EU27	GR	24.2	28.8	27.7	24.9	31.5	28.4	25.7	25	29.2	498	546	556	488
**Guyana** *		GY		43	55.7	55.6	55.7	43	55.7	55.6	55.7	1	1	1	1
**Hong Kong** *		HK	16.5	18	23.4	18	23.4	18	23.4	18	23.4	1	1	1	1
**Hungary**	EU27	HU	19	9.2	11.6	5.9	17.4	9.9	9.4	6.8	12.2	1589	1549	1660	1244
**Iceland**	Other Europe	IS	20	8.5	8	14.5	20.4	20	20	18.2	21.7	113	109	115	95
**India**		IN	33.5	27	27.2	21.9	33	31.1	27.5	23.4	34.1	1297	1279	1266	1094
**Ireland**	EU27	IE	12.5	16.1	15.4	14.3	19.1	13.2	12.9	12.4	14.2	926	904	729	549
**Italy**	EU27	IT	31.4	29.8	36.2	24.1	41.4	39.8	36.6	34.3	42.6	9880	10188	10886	8868
**Jamaica** *		JM	28.3	12.9	29		29	19.4	29		29	2	1		1
**Japan**		JP	37.2	29.7	36.2	28.6	39.2	39	41.3	36.4	43.4	7542	7241	7677	6836
**Kazakhstan**		KZ	20	19.1	25.3	16.3	28.2	21.7	21.4	19.9	24.3	57	56	58	54
**Kosovo** *	Other Europe	KV		10.7	12.9	10.2	13.9	12.6	13.8	12.2	14.5	2	2	2	2
**Latvia**	EU27	LV	15	9.5	12.1	8.6	13.8	15.7	15.2	15	16	809	823	847	784
**Lebanon** *		LB	15	16.1	16	15.8	16.2	16.1	16	15.8	16.2	1	1	1	1
**Liechtenstein** *	Other Europe	LI	12.5	12	13.2	9.6	18.2	12	13.2	9.6	18.2	1	1	1	1
**Lithuania**	EU27	LT	15	10.7	11.7	9.2	13.3	15.2	14.5	13.4	15.9	465	456	368	359
**Luxembourg**	EU27	LU	29.1	2.2	7.3	1.3	8.2	11.3	20.1	5.5	28.4	620	424	700	334
**Malaysia** *		MY	24.8	10				10				1			
**Malta**	EU27	MT	35	25	24.5	21.6	25.1	34.6	34.6	31.9	35	455	425	467	418
**Mauritius** *		MU	15	13.5	6.1	5.8	18.7	15.8	6.1	5.6	18.7	3	3	3	1
**Mexico**		MX	30	29.2	25.3	22.5	32.2	26.7	21.9	18.9	28.1	82	73	73	62
**Moldova** *	Other Europe	MD	12	11.9	11.5			9.6	10.1			8	7		
**Montenegro** *	Other Europe	ME	9	10.8	12.8	9.9	14.2	8.7	8.3	7.2	9.9	17	17	17	16
**Morocco** *		MA	30.3	31.3	29.3	26.2	35	24.9	23	21.2	24.8	47	46	49	44
**Netherlands**	EU27	NL	25	12.3	29.6	9.2	33.5	23.6	24	19.7	25.3	2205	1770	2028	1423
**New Zealand**		NZ	28	25.9	26	16.4	30.5	28.4	29.1	26.7	30.4	320	295	324	285
**North Macedonia**	Other Europe	MK	10	5.3	8.3	4.2	8.1	7.5	7.4	6.2	8.6	137	133	145	120
**Norway**	Other Europe	NO	27.6	52.6	62	46	67.1	27.7	27.5	23.7	29.7	4510	3890	4664	3557
**Pakistan** *		PK	34.4	34.2	30.3	29.7	33.7	31.5	30.4	29.6	32.2	27	26	24	25
**Panama** *		PA	25	30.3	26.4	25.9	31.1	29.8	26.4	25.6	30.8	4	4	4	4
**Paraguay** *		PY	10	36	24.4	23.7	37.6	36	24.4	23.7	37.6	1	1	1	1
**Peru** *		PE	30	32.5	30.7	27.1	37.6	31	27.7	24.7	34.6	37	33	35	32
**Philippines**		PH	30	21.8	20.4	20.4	21.2	30.1	28.5	28.4	29.7	552	570	571	559
**Poland**	EU27	PL	19	16.8	17.9	13.5	20.7	20.4	19.7	18.3	22.3	3851	3858	3819	3522
**Portugal**	EU27	PT	23.8	19.6	18	16.3	21.4	25.3	22.8	22.5	25.6	2890	2979	3011	2834
**Romania**	EU27	RO	16	17.4	16.7	13.1	21.6	17.2	16	12.8	20.7	2462	2542	2680	1936
**Russia**		RU	20	17.9	19.5	16.4	21.4	19.2	19	18.1	19.9	4443	4275	4452	4174
**Singapore**		SG	17	7.2				12.4				552			
**Slovakia**	EU27	SK	21	20.6	21.1	21.3	24.5	22.7	21.3	21.1	23.1	2539	2585	2614	2412
**Slovenia**	EU27	SI	17.8	13.9	13.7	11.6	16.1	17.3	16.4	14.7	18.6	870	853	850	768
**South Africa** *		ZA	30.6	16				16				1			
**South Korea**		KR	23.8	20.8	22.5	14.9	29.2	21.7	22.6	16.7	27.5	1483	1438	1527	1140
**Spain**	EU27	ES	29.6	20.7	22.9	16.4	27.4	28.1	26	23.4	30	7363	7012	7514	6580
**Sri Lanka** *		LK	28	29.5	31.5	27.6	33.9	11	20.1	9.4	22	16	15	17	14
**Sweden**	EU27	SE	23.7	13.3	18.3	11.1	20.8	20.7	20.7	18.4	22.7	6041	5515	5891	5038
**Switzerland** *	Other Europe	CH	18	9.4	5.8	5.1	8.2	20.9	15.1	14.8	23.7	30	26	27	24
**Taiwan** *		TW	17	22.1	18.7	18.6	23.9	18.4	19.7	18	21	12	11	12	11
**Thailand** *		TH	22.6	18	16.9	15.1	23.3	19.8	20.2	19.8	20.6	12	12	9	12
**Trinidad and Tobago** *		TT	25	26.4	25.9	25.8	27.2	27.8	26.7	25.4	29.7	2	2	2	2
**Tunisia** *		TN	28	14.3	7.4	7.3	14.9	14.4	7.4	7.2	14.9	2	2	2	2
**Turkey**		TR	20	16	13.9	6.9	23.5	20	15.2	7.9	29	293	295	307	152
**Ukraine**	Other Europe	UA	20.2	22	20.2	19.7	22.8	21.6	21	20.6	22.2	651	663	674	641
**United Arab Emirates** *		AE	55	96	94.7	94.7	96.1	96	94.7	94.7	96.1	1	1	1	1
**United Kingdom**	Other Europe	GB	22.8	13.7	22.7	12	22.9	22.1	22.4	19.6	23.8	10033	9004	8737	6011
**United States** *		US	40	9.3	8.9	8.9	10.2	9.3	8.9	8.9	10.2	1	1	1	1
**Uruguay** *		UY	25	3.6	3.8	3.7	4	17.6	16.3	14.3	18	26	28	30	22
**Uzbekistan** *		UZ		15.2	23.7	9.6		15.2	23.7	9.6		1	1	1	
**Serbia**	Other Europe	RS	13	7.7	7.3	5.7	10.3	10.7	9.6	7.4	13.4	680	680	713	583

Notes: Corporate income statutory tax rates (CIT), means and medians of ETRs in four estimations (ETR1–ETR4, defined in text) for 2011–2015. EU27 indicates whether a country was in the EU in February 2020 while Europe indicates non-EU countries geographically located mostly in Europe (i.e. this designation excludes Russia and Turkey). Countries marked with an asterisk (*) have fewer than 50 companies per sample. Source: Authors.

We propose two additional estimates to delineate the lower and upper bounds of true ETR values. We first estimate the lower bound, i.e., ETR3, by adding financial revenue to corporate income tax and operating profit—which constitutes the denominator of ETR2. Since financial revenue is always positive, the denominator is the highest of the four ETR versions, therefore producing the lowest ETR (as low or lower than the true ETR). Second, we estimate the upper bound, i.e., ETR4, by subtracting financial expenses from corporate income tax and operating profit—which constitutes the denominator of ETR2. Since financial expenses are always positive, the denominator is thus the lowest of the four ETR versions, therefore producing the highest ETR (as high or higher than the true ETR). Similarly to the point estimates, these supplementary bound estimates can be calculated as follows:

$${ETR3}_{it}=\frac{\sum {Corporate\;income\;tax}_{it}}{\sum {\left(Operating\;profit+financial\;revenue\right)}_{it}}$$

$${ETR4}_{it}=\frac{\sum {Corporate\;income\;tax}_{it}}{\sum {\left(Operating\;profit-financial\;expenses\right)}_{it}}$$

Overall, having considered alternative approaches to address the above-mentioned data limitation, we opt for the inclusion of four ETR versions in the absence of one true ETR value estimate. Since ETR1 and ETR2 are both candidates for the most suitable point estimate of the true ETR, and since no overriding argument in favour of one or the other exists, we include both. We include ETR3 and ETR4 because they serve as lower and upper bounds, thus indicating the range, however wide, where the true ETR is to be expected with confidence. Furthermore, by not including financial revenue in the denominator, only ETR2 and ETR4 are not vulnerable to the potential double counting of profit for holding companies in particular, which are concentrated (and therefore more likely to skew results) in some countries such as the Netherlands and Luxembourg (on potential double counting see discussions by [21, 50, 54, 55]. Although we value the simplicity of one ETR version, in the face of the complexity of available data we include four complementary versions, presented alongside each other in the results below. In addition to using mean values in our headline estimates, we provide results following the same methodology, but applying medians instead of means (different indicators of distribution may be provided as well, though we do not present these due to limited space). Using median values ensures that the results are less affected by large companies. For most countries the median values are higher than means (which is consistent with recent research by [56], who show that in particular large companies engage in profit shifting). While for our paper we present mean values as headline estimates, as is common in existing literature, median-based estimates may be useful for other research questions in the future.

We aim to utilize as much data as possible for estimating each of the four ETR versions. In doing so, we use all data available for the estimation for each of the four versions, i.e., we use a different data sample for each version due to differences in the availability of individual variables. In general, operating profit data are available for more companies than financial profit data; furthermore, financial profits are not divided into financial expenses and revenue for some companies in some countries. Of the 47 countries, there are two extreme examples. Estonia has over 500 companies with data available for ETR1 and ETR2 no company with data suitable for ETR3 and ETR4 while Singapore has over 500 companies with data available for ETR1 but no companies for any of the other ETR versions. In some countries, ETR4 estimates may thus not be the highest values of the four ETRs. This is a consequence of differences in utilized data samples rather than the outcome of inconsistencies in the design of the four ETRs. Alternatively, in contrast with using as much data as possible, and consequently utilizing different data samples for each of the four ETRs, it is possible to establish a single data sample with available data for all four ETR versions. While this alternative produces four empirically consistent ETR versions, each is based on a lower number of companies (e.g. in the case of Estonia all four versions are based on a single company) and it thus fails to exploit all of the available data. In the interests of providing a truly comprehensive approach, we implement this alternative as one of the robustness checks and present the results in the S1 Appendix.

ETRs estimated using unconsolidated data enable us to study the extent to which ETRs differ across countries and from their statutory rates. To compare these, we use one headline statutory rate for each country, which, of course, provides us with only an imperfect comparison, especially in case a given country implements a variety of rates either across various parts of the country (e.g. municipalities in Germany) or for different types of companies (e.g. Norway). In the case of Germany, research interest has recently surged, in part in response to this paper’s preliminary results used in a policy report [57] which sheds further light on why the estimated ETR range is rather wide (see e.g. in German literature [58, 59]). Germany is, of course, only one relatively well studied example, another is South Korea’s progressive tax schedule [60]. Indeed, many other countries exhibit specific corporate income tax complexities which co-determine the value of ETRs and generally make them lower than statutory rates.

More generally, when compared with statutory rates, ETRs might indicate the effect of tax deductions–including tax holidays and other arrangements such as tax exemptions and tax rulings–as well as other tax provisions which codetermine tax paid by companies and how ETRs differ across countries. For example, if an MNC affiliate receives a tax holiday or a tax ruling in a given country, we expect its ETR to be lower than the headline statutory rate. Some of these tax deductions and other regulations are captured both by backward-looking ETRs, which form the focus of this paper, and by forward-looking ETRs [10] recently estimated by the OECD [24], which we briefly compare our estimates to below. In contrast, we are not aware of recently available estimates of backward-looking macroeconomic approach, e.g. by [61], and therefore do not include them in our comparison.

To better understand these kinds of country-specific details related to corporate income taxation, we include Table A5 in the S1 Appendix with the characteristics that are more likely to be consequential for the estimated ETRs and their differences with statutory rates. For each country, the table lists the characteristics of the corporate income tax systems that differ across various parts of the country (e.g. states in the United States), for different types of companies (e.g. progressive taxation in South Korea) or other atypical features that define the tax rate or the taxable base differently from the standard corporate tax. We provide these characteristics for countries that have more than 50 companies per sample (i.e. not marked with an asterisk (*) in the results tables) and therefore with more reliable estimates of ETRs. For the sake of length and clarity, the table is not supposed to be comprehensive but highlights the most important examples, if any, for each country. These country-specific examples should make the reader aware of the intrinsic limitations of our proposed methodology, as well as any other cross-country empirical work analysing taxation using the Orbis data.

The question of how much taxes–and where–MNCs’ affiliates pay is best answered by examining ETRs estimated using unconsolidated data. Alternatively, to the extent that the worldwide taxation of MNCs headquartered in a given country is of interest, ETRs using consolidated MNC data may be estimated as a weighted average of company-level ETRs of companies headquartered in that country, as recently carried out by [33], but not to analyse ETRs of individual MNC affiliates. Ultimately, we consider both consolidated and unconsolidated data useful for estimating ETRs, though opting for one or the other is best governed by the purpose at hand. One natural consideration is data availability, which is generally better for consolidated rather than for unconsolidated Orbis data. Indeed, as we discuss above, unconsolidated data availability in terms of both coverage and quality limits the estimation of unconsolidated ETRs. Still, if one is interested in the overall effects of the corporate income tax systems of individual countries, these ETRs facilitate an unparalleled view of MNCs’ taxation.

## 4. Results

**Fig 1:**
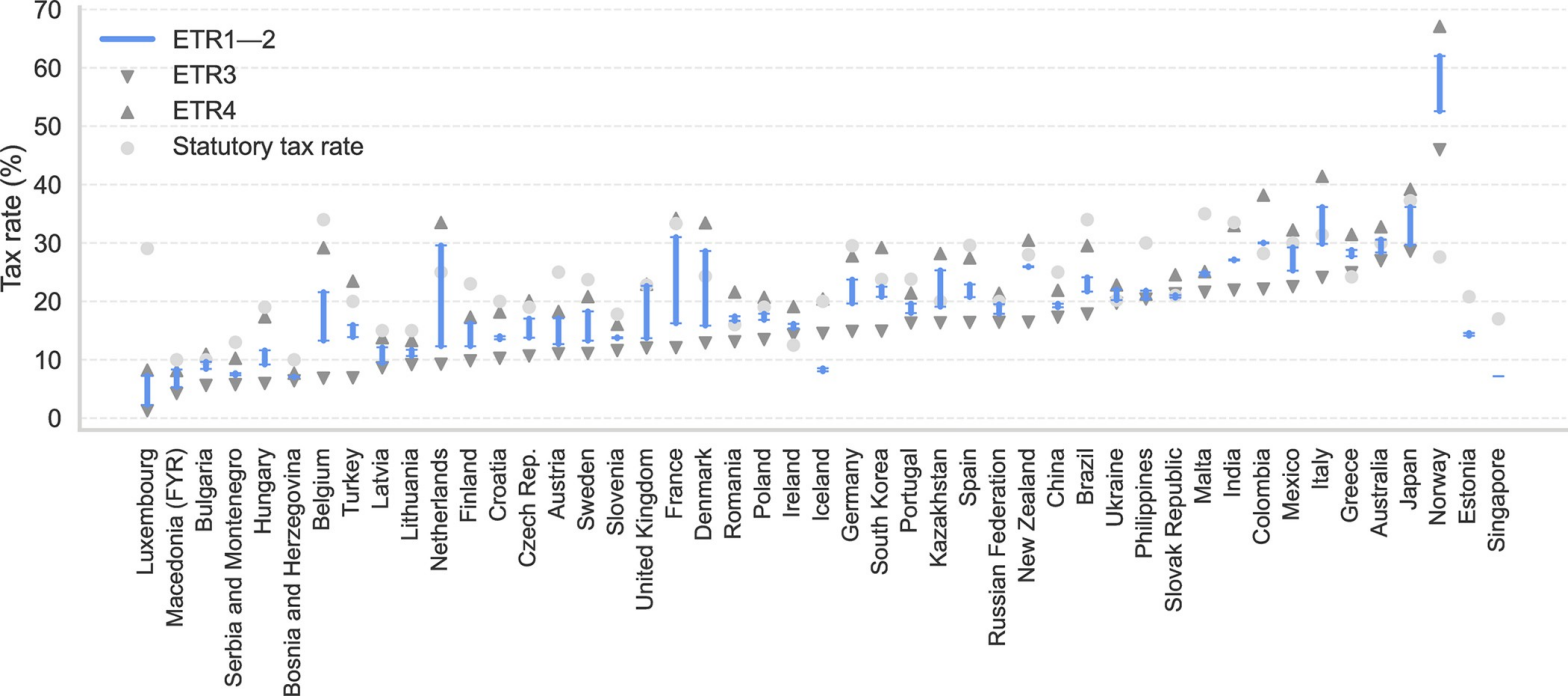
Effective tax rates. Notes: Corporate income statutory tax rates (CIT), means and medians of ETRs in four estimations (ETR1–ETR4, defined in text) for 2011–2015, sorted by ETR3. Countries with fewer than 50 companies per sample are not included in this figure. Source: Authors.

**Fig 2:**
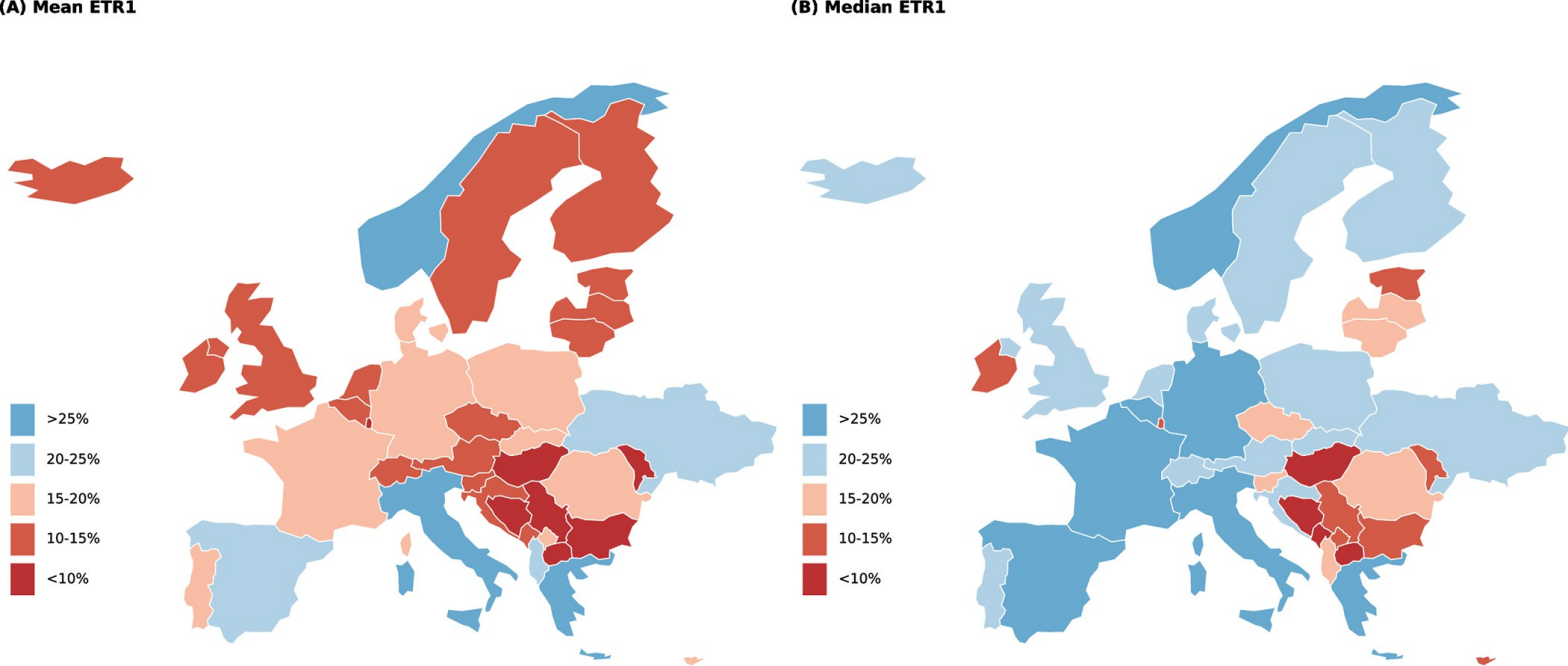
Effective tax rates in Europe. Notes: Mean ETR1 and ETR2 values in Europe. Countries with fewer than 50 companies per sample are not included in this figure. Source: Authors.

We present the results of ETRs of MNCs’ affiliates for individual countries calculated using Orbis data as gross income-weighted means for a five-year period between 2011 and 2015. First, we show the main results in Fig 1, which displays the four ETR versions described in Section 3 Methodology along with the applicable statutory rates (means for the 2011–2015 period). Fig 1 shows 47 countries with data on at least 50 companies, including all 27 EU member states as of February 2020 that are also shown on a map in Fig 2. In addition to providing the same results as Fig 1 and Table 2 includes estimates for all 93 countries with at least one company included in the data (with countries with fewer than 50 companies marked with an asterisk *) as well as the number of companies used to calculate each ETR version. This is provided for the sake of completeness and transparency, though results for countries with a limited number of companies should be treated with a higher degree of caution than the sample of 47 countries. Furthermore, in addition to means in Fig 1, we include estimates for medians in Table 2, which are more consistent across ETR1 to ETR4, but we use means in our headline estimates as is common in the existing literature. We now present some of the most interesting findings, mostly using the two extreme ETR values (i.e. ETR3 and ETR4) to describe the results.

We find that MNCs do not pay much tax on their profits in some countries. In four of the 47 countries included in Fig 1 (Luxembourg, North Macedonia, Bosnia and Herzegovina, and Serbia) we observe that all ETR values are lower than 10%. While our approach does not enable us to say whether this is what MNCs should be paying, some of these values are low both absolutely and in contrast to statutory rates (in the case of Luxembourg) as well as in comparison to other countries (Bosnia and Herzegovina, North Macedonia, and Serbia). A case in point is Luxembourg with a lower bound (ETR3) of 1% and an upper bound (ETR4) of 8%, but a statutory rate of 29%. Some evidence indicates that tax rulings have played a role in Luxembourg’s low ETR [60].

Differences in ETRs were observed between individual countries. Out of the 47 countries and across the four ETR versions displayed in Fig 1, MNCs may expect to pay between 0% and 10% in 5 countries, 10–20% in 20 countries, 20–30% in 20 countries, and over 30% in 5 countries (and as little as 1% or as much as 65% in the most extreme cases) of their profit in taxes (numbers of countries are approximate due to the four versions of ETRs). Fig 1 confirms that Bosnia and Herzegovina, North Macedonia, and Luxembourg (the first two non- EU; with non-EU Serbia and Turkey having low ETRs, too) have the lowest ETR (with all four ETR versions below 10%) of the 50 displayed countries while Norway has the highest ETR (ETR3: 45%; ETR4: 66%). Most other countries with high ETRs are generally non-European countries such as Japan, Peru, Australia, or Morocco. In the EU, in addition to Luxembourg, countries with the lowest ETRs include Cyprus (7–11%) and Bulgaria (6–11%). While other EU member states have relatively low lower bounds, their upper bounds tend to be higher, e.g. in the case of Hungary (6–18%), Belgium (8–31%) and the Netherlands (10–34%). EU countries with the highest ETR include Italy (25–42%) and Greece (24–31%). The ETRs of the remaining 20 EU countries range between 10% and approximately 30%, including those of the biggest EU economies such as France (13–35%) and Germany (15–29%). The United Kingdom (13–24%) falls into the same range, though it is no longer an EU member state.

### 4.1 Effective tax rates and statutory rates

As expected, ETRs are lower than statutory rates in most countries. ETRs 1, 2, 3 and 4 are lower than statutory rates in 40, 37, 43 and 23 out of the 47 countries. This is natural in view of tax holidays and other tax provisions, which lead to ETRs being lower than statutory rates.

Still, there are countries for which at least some estimated ETRs are higher than statutory rates. Specifically, ETRs 1, 2, 3 and 4 are higher than statutory rates in 7, 10, 4 and 24 out of the 47 countries. On the one hand, only three countries, which we discuss below, have the lower bound ETR (ETR 3) higher than statutory rates. On the other hand, the number of countries in which other ETR estimates are higher than statutory rates is not negligible and does not enable us to investigate them empirically case-by-case. Instead, we outline two broad and interrelated reasons why ETR estimates are higher than statutory rates–tax system and data characteristics. First, corporate taxation is often more complicated than implicitly assumed in our straightforward comparison of ETRs and statutory rates and we highlight some of the most important country-specific details in Table A5 in the S1 Appendix, ranging from subnational tax rates to industry-specific tax provisions. Second, as discussed in Section 2 Data, the data has a number of limitations, including the fact that the quality and granularity of the data does not enable us to take into account the country-specific details in the ETR estimations.

Lower ETR bounds, i.e. ETR3, are higher than statutory rates only in Norway, Ireland and Estonia and we discuss each of them now. We observe the only substantial difference in the case of Norway, where the four ETR versions are 53%, 62%, 46% and 67%, whereas the statutory rate of 28% is far lower. This is likely due to special tax provisions applicable to the Norwegian petroleum sector, which is subject to a marginal tax rate of up to 78% [62]. Ireland, with ETR values of 16%, 15%, 14% and 19% and a statutory rate of 12.5%, presents a more intriguing case; however, given the data limitations, these results are consistent with existing literature. Our data does not account for the case of the Apple company [21] and the Irish public audit body has recently found ETRs of similar magnitude [63]. The audit body also counters the evidence of low taxation in Ireland presented by e.g. [64] by arguing that the Bureau of Economic Analysis data used for this approach includes financial data from MNCs’ operations everywhere, not just in Ireland, and, as such does not necessarily constitute a reflection of MNCs’ operating activities in Ireland or corporation tax paid in Ireland [63]. Estonia is a very special case, because the estimates of ETR3 and ETR4 are based on an observation of only one company in the Orbis data (in contrast with over 600 companies with data available for ETR1 and ETR2). Our use of Orbis-based ETRs for Ireland and other countries is further supported by the fact that they are positively, albeit of course not perfectly, correlated with backward-looking ETRs based on other data sources including country-by-country reporting data for US-headquartered MNCs [49].

Furthermore, we observe sizeable differences between individual countries with respect to how much lower ETRs are than statutory rates. On the one hand, countries such as Ukraine, Bulgaria, and Slovakia exhibit ETRs comparable to their statutory rates. In the case of these countries, the statutory rate provides approximate information on corporate income tax which MNCs can expect to pay. On the other hand, substantial differences between the two rates are found in a range of other countries. While Luxembourg once again constitutes a case in point, statutory rates do not provide a great deal of information on the tax burden MNCs face in many other countries.

ETRs and statutory rates are positively related, though less so in the case of EU countries (Fig A1 in the S1 Appendix). At country level, the correlation between the four ETR versions and statutory rates is 0.58, 0.63, 0.51, and 0.64 (estimates for our sample of 47 countries which includes some non-EU countries while facilitating a comparison between EU countries and the rest of the world; estimates for a sample of 93 countries are similar: 0.53, 0.55, 0.52, and 0.52). However, in the case of EU countries, the correlation is only similar for ETR2 and ETR4, with ETR1 and ETR3 values being at approximately one half: 0.37, 0.64, 0.28 and 0.61. While statutory rates may thus be viewed as informative with respect to worldwide ETRs, they tend to be less informative in the case of EU countries–but this is likely driven by the above discussed case of Luxembourg (indeed, when we exclude Luxembourg as a robustness check, the differences in correlation between the main sample and the EU sample are no longer present). Similarly, forward-looking ETRs are positively correlated with our ETRs, and forward-looking ETRs are also included for comparison (with mostly higher values than our backward-looking ETRs) in Fig A2 in the S1 Appendix and Table 2.

### 4.2 Robustness checks

In addition to the main results provided in Fig 1 and Table 2, we present the results of five robustness checks which are broadly consistent with our main results. The results of the first four of these robustness checks are presented in the Tables A1-A4 of the S1 Appendix. In Table A1 in the S1 Appendix we do not exclude observations for companies with negative profits in the previous year. In Table A2 in the S1 Appendix we use a balanced panel data set for all four ETR versions. In Table A3 in the S1 Appendix we apply both these conditions simultaneously. In Table A4 in the S1 Appendix we keep only affiliates with 3 or more observations between 2011 and 2015.

Fifth, we perform a sectoral robustness for two reasons. Countries may have some sectors that are large and have sector-specific taxation and therefore the ETR estimated for such a country might not be representative of other sectors (e.g., oil in Norway, as discussed above). This kind of heterogeneity can be especially important for countries with few observations, where those few might come disproportionally more often from a specific sector in which ETRs are particularly high or low. Also, the intensity of tax avoidance is different across sectors, which would also affect computed ETRs. If any of the two conditions are true, then the estimated ETRs would become less informative about ETRs of all MNCs active in the country. Furthermore, the Orbis dataset might include some bias, and over- or under-represent sectors in some countries.

To address these concerns for sectoral heterogeneity, specifically, we eliminate one sector (using the NACE 1-digit classification) at a time from the analysis. Each graph in Fig A3 in the S1 Appendix shows the robustness check for the mean ETR (one graph for each type of ETR). The ETRs are calculated for every country for all companies excluding one sector at a time. The ETR when a sector is removed increases or decreases by more than 5 percentage points in 28 instances across all countries and types of ETRs and only for four sectors. These four sectors are, in the alphabetical order as well as according to the frequency: B (Mining and Quarrying; 15 cases across the four types of ETRs), C (Manufacturing; 6 cases), K (Financial and Insurance Activities; 5 cases), N (Administrative and Support Service Activities; 2 cases). The complete list of NACE codes is in the Table A6 in the S1 Appendix. While taxation of mining often takes special forms [65], financial and insurance activities and administrative and support service activities are among the sectors that are viewed as vulnerable to profit shifting strategies [38].

The results are in general very robust to exclusion of individual sectors, except for Norway (excluding mining, in which the oil industry is included, the tax rate decreases to around 16%) and a few other cases. For example, the mining sector also increases the tax rate by more than 5 percentage points in some other countries such as Denmark and the Netherlands. We also observe that the financial sector reduces greatly the tax rate for ETR1 and ETR3 in the Netherlands and Cyprus. Overall, these are individual exceptions and the results are in general robust to excluding individual sectors.

## 5. Conclusions

While the then OECD Secretary-General Angel Gurría stated that the ultimate goal is ensuring that all MNCs “pay their fair share” [66], there is currently no consensus regarding the amount of corporate income tax paid by MNCs in individual countries. In this paper, our aim is to contribute to creating such consensus. We proceed in three steps. First, we argue that ETRs estimated from the income statement data of MNCs are useful for comparing MNCs’ corporate income taxation across countries. Second, we propose a new methodological approach to estimate ETRs as reliably and for as many countries as possible. Third, we apply the new methodology to Orbis’ unconsolidated data to arrive at new results for 50 mostly European countries. We find that ETRs substantially differ across countries and from statutory rates for some countries. These findings should be of particular interest in the light of recent changes in the taxation of MNCs worldwide such as the 2021 agreement of more than 100 countries worldwide to a global minimum tax rate of 15%, so called Pillar 2. Indeed, to discuss the effects of any reform of the taxation of MNCs, we first need to establish the state of play, starting with how much tax MNCs pay.

To estimate ETRs of MNCs in individual countries, we use the best available company-level data for many countries, mostly in Europe. While the Orbis database does constitute the best available source, which thus enables us to study how much ETRs differ across countries or from country-specific statutory rates, it suffers from several inherent shortcomings, including the fact that its income statement data does not differentiate between equity income and other types of financial income. Better data are needed in order to achieve more informed policy decisions and obtain more reliable ETR estimates. One source of better data could come in the form of public, affiliate-level country-by-country reporting data, i.e. in contrast with aggregate data from tax authorities on the biggest MNCs, which the OECD has published since 2020. Results obtained using such data would likely be even more suitable for follow-up research than those achieved using Orbis, e.g., when examining differences in ETRs between individual MNCs and across various fields.

The estimates of MNCs’ ETRs allow us to establish differences in observed ETRs across countries. In many countries MNCs do not pay much tax. As expected, ETRs are lower than statutory rates in many countries, but countries differ significantly with respect to how much. ETRs and statutory rates are positively related, though less so in the case of EU countries. Presented evidence suggests to some extent a race to the bottom in ETRs: some EU countries do not tax MNCs much and that these EU countries cannot lower their rates further since they are already close to the bottom. Furthermore, as some of these very same EU member states are using their rights to block some of the tax reforms discussed at EU level, the EU could abandon the requirement for unanimity in tax matters and the European Commission should consider using Article 116 of the Treaty on Functioning of the European Union to propose legislation in this respect. This would be especially in case the implementation of the two-pillar solution would stall for some reason, although the adoption of Pillar 2 as mandatory for all EU member states through an EU Directive in December 2022 is encouraging. Indeed, the EU’s leadership could incentivise other countries to implement Pillar 2, too. Furthermore, the EU member states as well as other countries should consider the adoption of Pillar 1 or other reforms, designed to reduce the profit shifting and the unhealthy tax competition practices.

The new methodology and estimates of MNCs’ ETRs should also inform future research. There are fundamental research questions that can be tackled using the approach, especially when it is advantageous that the ETRs are for MNCs specifically or that the ETRs are backward-looking. A first example of such a question is how corporate taxation influences investment. Many studies examine how tax rates in a given country affect investment in that country or reallocation of investment to or from other countries (e.g., a meta-analysis by [67]). Often for the lack of a better alternative, most of these studies use statutory rates or forward-looking ETRs. We contribute to improving the availability of backward-looking ETRs, which can substantially differ, as in the case of Luxembourg (our estimates of 1–8% in contrast to statutory rate of 28% and forward-looking ETRs above 20% according to [24]. A second example is the profit shifting literature (e.g., a meta-analysis by [68], which has historically mostly relied on statutory rates with one of the reasons against ETRs being their lack of availability, which we address and future research can build on by providing, for example, estimates of ETRs over time.

Another actionable implication for future research relates to the already discussed global minimum tax reform to be implemented in many countries worldwide and our methodology should be useful in evaluating the reform’s effects ex ante as well as ex post. And there are other similar research questions and many tax and public economics researchers seem to be interested in using MNCs’ ETRs as control variables. Which corporate rate is advisable to use will always require an individual careful consideration, but we naturally consider improving the breadth and quality of the options from which researchers can choose, as we do in this paper, a welcome development.

## Supporting information

S1 Appendix Additional results. (DOCX)
