# Supplementary material for: Effective tax rates of multinational corporations: Country-level estimates
Source: PLoS One. 2023 Nov 29;18(11):e0293552. doi: 10.1371/journal.pone.0293552 (PMC10686448; doi:10.1371/journal.pone.0293552)
Supplement: S1 Appendix — (DOCX) [file pone.0293552.s001.docx]

# Appendix

**Figure A1. Correlation between effective tax rates**

| 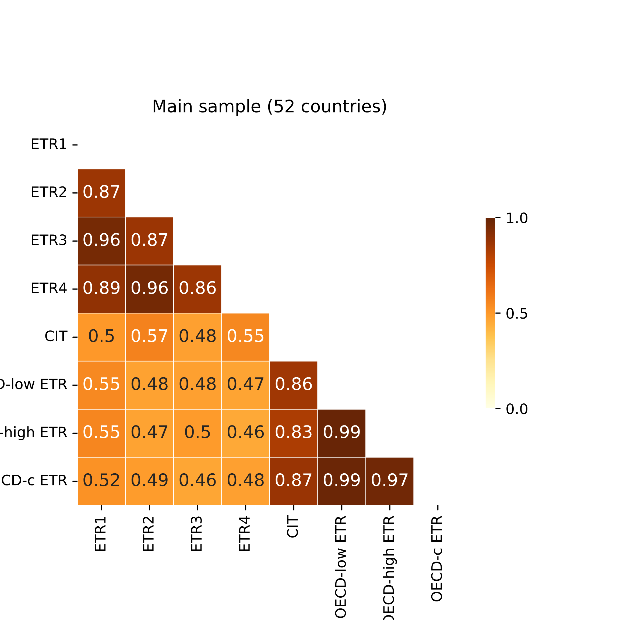 | 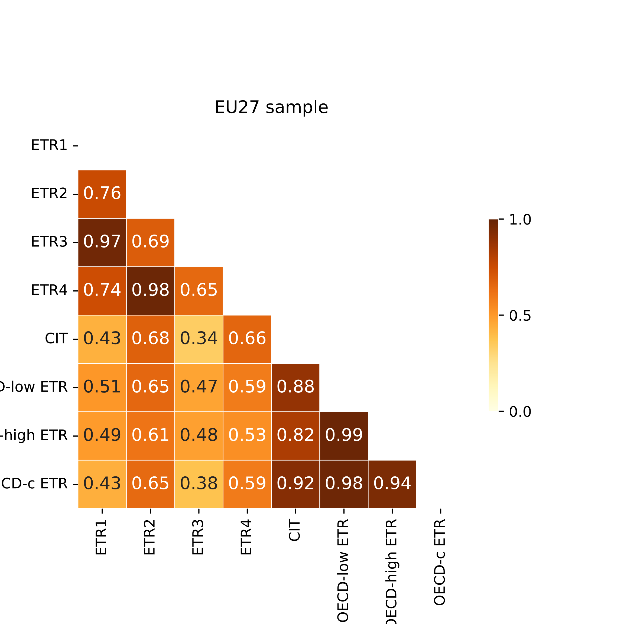 |
| --- | --- |

*Notes: Corporate income statutory tax rates (CIT), means and medians of ETRs in four estimations (ETR1–ETR4, defined in text) for 2011–2015.* *OECD forward-looking ETRs (dataset CTS_ETR) for three scenarios: high inflation and interest rates (OECD-high), low inflation and interest rates (OECD-low) and country-specific inflation and interest rates (OECD-c). Source: Authors.*

**Figure A2. Effective tax rates, including additional data sources**


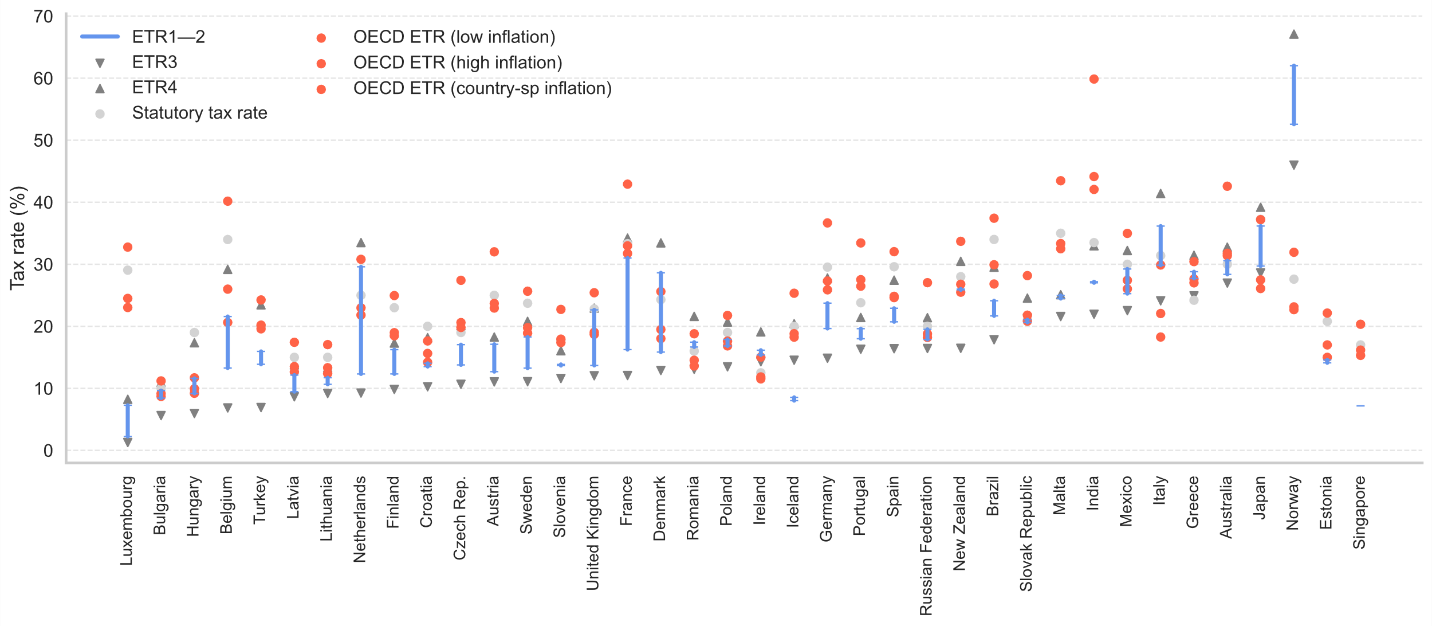


*Notes: Corporate income statutory tax rates (CIT), means and medians of ETRs in four estimations (ETR1–ETR4, defined in text) for 2011–2015.* *OECD forward-looking ETRs (dataset CTS_ETR) for three scenarios: high inflation and interest rates (OECD-high), low inflation and interest rates (OECD-low) and country-specific inflation and interest rates (OECD-c). Source: Authors.*

**Figure A3** **.** **Effective tax rates, robustness checks – excluding one sector at a time**

**
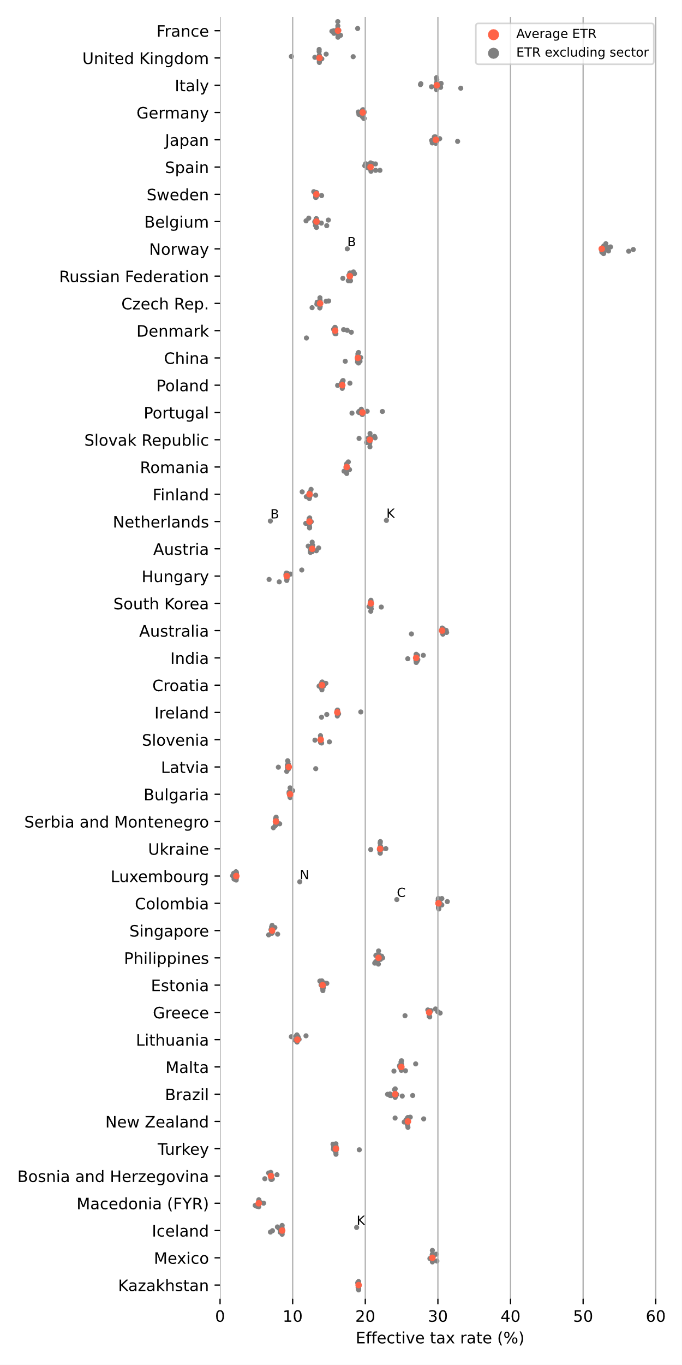
**
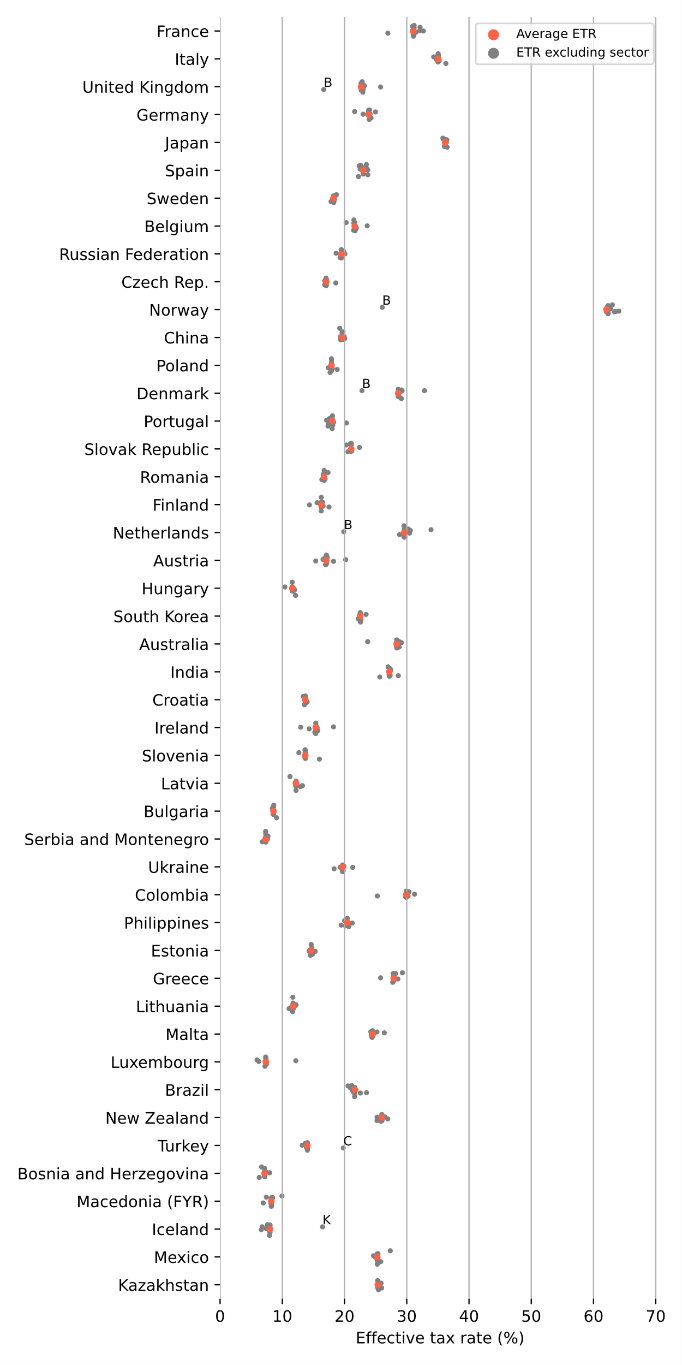


Panel 1: ETR1 Panel 2: ETR2

***
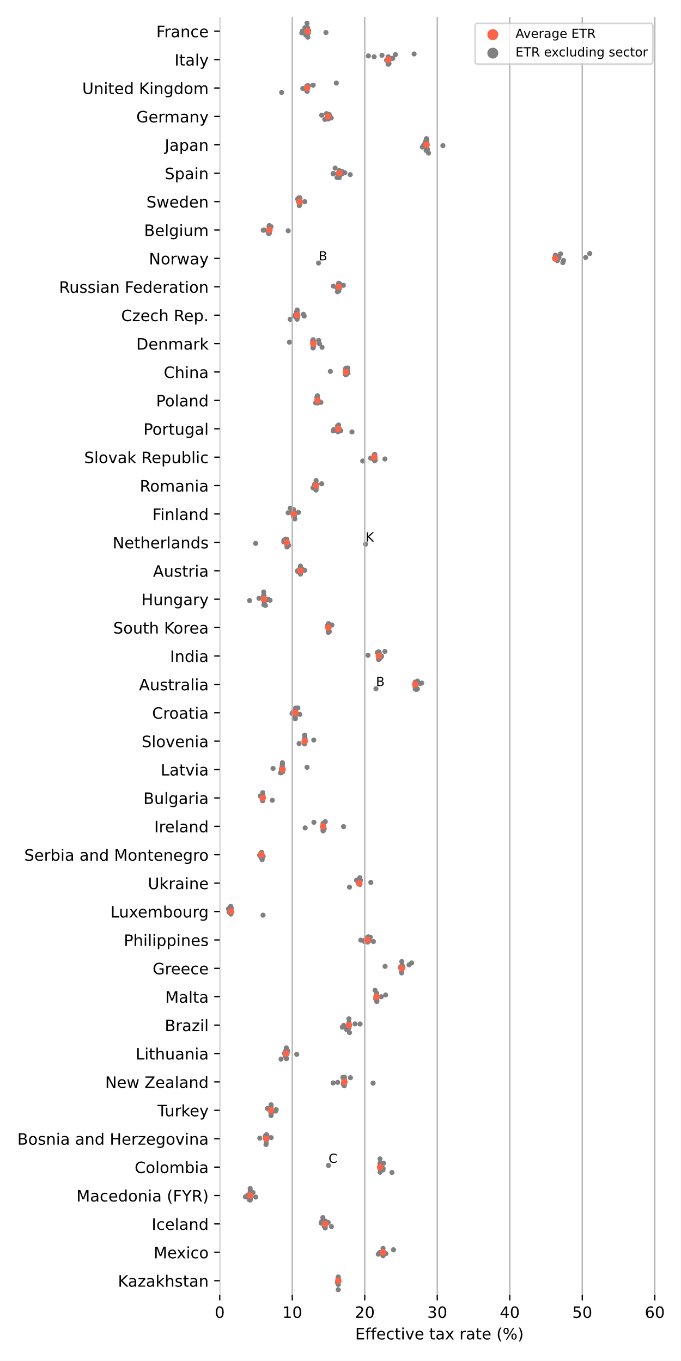
***
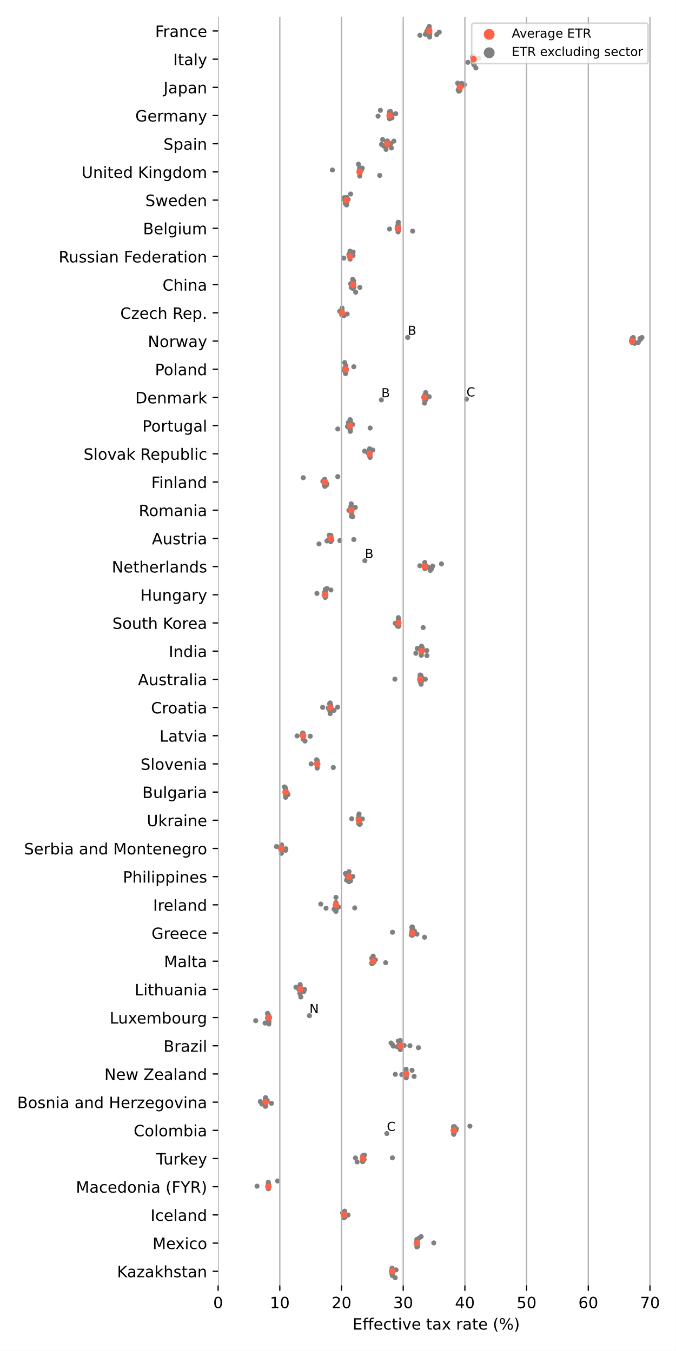


*Panel 3: ETR3 Panel 4: ETR4*

*Notes: Each graph shows the robustness test for the mean ETR (one graph for each type of ETR). The ETRs are calculated for every country for all companies excluding one sector at a time. Each dot represents the mean ETR without one sector. The orange dot is the average ETR for all sectors, while the grey dots represent the mean ETR without one sector at a time. When removing the sector increased or decreased the country's ETR by more than 5 percentage points, the dot is labelled with a letter representing the sector’s NACE code. This occurs only for four sectors, in the alphabetical order as well as according to the frequency: B (Mining and Quarrying; 15 cases across the four types of ETRs), C (Manufacturing; 6 cases), K (Financial and Insurance Activities; 5 cases), N (Administrative and Support Service Activities; 2 cases). The complete list of NACE codes is in the Table A6 in the Appendix. Source: Authors.*

**Table A1. Effective tax rates, robustness check (not removing observation when a company had negative profits in the previous year)**

| Country name | EU | ISO2 | CIT (%) | | Mean (%) | | | | | | | Median (%) | | | | | Number of companies | | | |
| --- | --- | --- | --- | --- | --- | --- | --- | --- | --- | --- | --- | --- | --- | --- | --- | --- | --- | --- | --- | --- |
|  |  |  |  |  | **ETR1** | **ETR2** | | **ETR3** | | **ETR4** | | **ETR1** | | **ETR2** | **ETR3** | **ETR4** | **ETR1** | **ETR2** | **ETR3** | **ETR4** |
| Albania* | Other Europe | AL | | 12 | 22.6 | | 23.2 | | 21.8 | | 26.5 | | 15.8 | 15.6 | 15.4 | 16.1 | 31 | 30 | 30 | 29 |
| Algeria |  | DZ | | 25 | 25.2 | | 20 | | 18.3 | | 22.9 | | 24.9 | 23.5 | 22.6 | 25.8 | 133 | 132 | 136 | 118 |
| Argentina |  | AR | | 35 | 34.2 | | 35.5 | | 31.5 | | 44.5 | | 35.1 | 33.9 | 31.6 | 37.5 | 63 | 49 | 50 | 35 |
| Armenia* |  | AM | | 20 | 22 | |  | |  | |  | | 22 |  |  |  | 1 |  |  |  |
| Australia |  | AU | | 30 | 28.8 | | 26.6 | | 25.2 | | 30.8 | | 30 | 29.2 | 27.7 | 30.9 | 2482 | 2427 | 2134 | 1770 |
| Austria | EU27 | AT | | 25 | 11.9 | | 15.2 | | 10.1 | | 16.9 | | 21.3 | 22.4 | 17.9 | 24.9 | 2509 | 2297 | 2494 | 2022 |
| Barbados* |  | BB | | 25 | 1.8 | |  | | 1.8 | |  | | 1.8 |  | 1.8 |  | 1 |  | 1 |  |
| Belarus* |  | BY | | 19.2 | 18.5 | |  | |  | |  | | 39.9 |  |  |  | 2 |  |  |  |
| Belgium | EU27 | BE | | 34 | 12.5 | | 21.8 | | 7 | | 29.2 | | 27.7 | 28.7 | 21.4 | 34.1 | 8329 | 7509 | 8327 | 6750 |
| Bermuda* |  | BM | | 0 | 18.6 | | 36.8 | | 16.8 | | 46.5 | | 18.6 | 36.8 | 16.8 | 46.5 | 1 | 1 | 1 | 1 |
| Bolivia* |  | BO | | 25 | 24.9 | | 12.8 | | 3 | | 14.5 | | 19.8 | 12.5 | 3 | 13 | 3 | 2 | 1 | 2 |
| Bosnia and Herzegovina | Other Europe | BA | | 10 | 7.1 | | 7.2 | | 6.5 | | 7.8 | | 9.8 | 9.5 | 8.8 | 10.3 | 308 | 296 | 309 | 285 |
| Brazil |  | BR | | 34 | 25.3 | | 23.6 | | 15.1 | | 28.9 | | 29.5 | 22.6 | 19 | 32.6 | 740 | 654 | 714 | 530 |
| Bulgaria | EU27 | BG | | 10 | 9.8 | | 8.7 | | 6.2 | | 11 | | 10.1 | 9.7 | 9.3 | 10.6 | 1096 | 1032 | 1099 | 986 |
| Burkina Faso* |  | BF | | 16 | 32.9 | | 30 | | 29.7 | | 33.3 | | 32.9 | 30 | 29.7 | 33.3 | 1 | 1 | 1 | 1 |
| Cambodia* |  | KH | | 20 | 4.6 | |  | |  | |  | | 4.6 |  |  |  | 1 |  |  |  |
| Canada* |  | CA | | 26.6 | 27.1 | | 35.7 | |  | |  | | 27.2 | 35.7 |  |  | 3 | 1 |  |  |
| Cape Verde* |  | CV | | 25 | 24.5 | | 22.1 | | 21.6 | | 25.2 | | 24.5 | 22.1 | 21.6 | 25.2 | 1 | 1 | 1 | 1 |
| Chile* |  | CL | | 20.5 | 20.8 | | 18.8 | | 16.1 | | 23.9 | | 18.4 | 16.7 | 13.6 | 21.7 | 37 | 34 | 35 | 32 |
| China |  | CN | | 25 | 19.3 | | 19.4 | | 17.5 | | 21.7 | | 22.2 | 20.7 | 19.8 | 23.6 | 7242 | 7080 | 7197 | 6964 |
| Colombia |  | CO | | 28.2 | 29.7 | | 29.1 | | 21.4 | | 37.4 | | 33.2 | 32.8 | 27.5 | 37.8 | 845 | 845 | 271 | 222 |
| Costa Rica* |  | CR | | 30 | 37.5 | |  | |  | |  | | 37.5 |  |  |  | 1 |  |  |  |
| Cote d'Ivoire* |  | CI | |  | 20.6 | | 21.2 | | 18.7 | | 23.6 | | 20.8 | 25 | 20.7 | 25.8 | 3 | 3 | 3 | 3 |
| Croatia | EU27 | HR | | 20 | 14.6 | | 14.5 | | 11.2 | | 17.9 | | 20.4 | 19.6 | 17.3 | 22 | 1179 | 1130 | 1195 | 1019 |
| Cyprus | EU27 | CY | | 11.5 | 17.7 | | 10.7 | | 10.6 | | 12.7 | | 12.1 | 10.9 | 9.5 | 12.5 | 163 | 158 | 109 | 136 |
| Czechia | EU27 | CZ | | 19 | 14 | | 16.8 | | 10.7 | | 20 | | 19.4 | 18.4 | 15.8 | 21.9 | 5576 | 5424 | 5657 | 4734 |
| Denmark | EU27 | DK | | 24.3 | 15 | | 27.1 | | 12.2 | | 31.6 | | 24.3 | 24 | 20.4 | 26 | 5428 | 4482 | 5265 | 4097 |
| Dominica* |  | DM | |  | 28.2 | | 22.4 | |  | | 28.2 | | 28.2 | 22.4 |  | 28.2 | 1 | 1 |  | 1 |
| Ecuador* |  | EC | | 22.6 | 26.9 | | 25.2 | | 23 | | 28 | | 26.8 | 22.7 | 21.2 | 26.9 | 14 | 14 | 12 | 14 |
| Egypt* |  | EG | | 23.5 | 50.5 | | 57.5 | | 57 | | 57.9 | | 22.1 | 33.4 | 33.1 | 34.7 | 4 | 2 | 2 | 2 |
| El Salvador* |  | SV | | 30 | 50.2 | | 40.1 | | 22.3 | | 58.1 | | 42.7 | 30.5 | 23.5 | 54.7 | 3 | 3 | 2 | 2 |
| Estonia | EU27 | EE | | 20.8 | 12.3 | | 14.7 | |  | |  | | 14.7 | 15.1 |  |  | 853 | 833 |  |  |
| Finland | EU27 | FI | | 23 | 12.3 | | 16.1 | | 9.8 | | 17.2 | | 21.5 | 21.2 | 19.7 | 22.9 | 3417 | 3254 | 3266 | 2978 |
| France | EU27 | FR | | 33.3 | 16.3 | | 30.6 | | 12.2 | | 33.8 | | 28.2 | 28.6 | 25.1 | 31.1 | 17092 | 15702 | 17256 | 14821 |
| Gabon* |  | GA | |  | 15.9 | | 14.9 | | 14.6 | | 15.8 | | 22.4 | 18.5 | 18 | 21.4 | 2 | 2 | 2 | 2 |
| Germany | EU27 | DE | | 29.5 | 19.6 | | 23.1 | | 14.8 | | 26.5 | | 26.5 | 25.1 | 20.8 | 29.6 | 11782 | 10863 | 11353 | 9540 |
| Greece | EU27 | GR | | 24.2 | 28 | | 26.2 | | 24 | | 30.3 | | 28.8 | 26.2 | 25.5 | 29.6 | 631 | 649 | 660 | 617 |
| Guyana* |  | GY | |  | 43 | | 55.7 | | 55.6 | | 55.7 | | 43 | 55.7 | 55.6 | 55.7 | 1 | 1 | 1 | 1 |
| Hong Kong* |  | HK | | 16.5 | 17.3 | | 22.2 | | 17.3 | | 22.3 | | 10.1 | 12.8 | 10.1 | 12.9 | 2 | 2 | 2 | 2 |
| Hungary | EU27 | HU | | 19 | 8.1 | | 10.8 | | 5.5 | | 15.2 | | 9.8 | 9.3 | 6.7 | 11.6 | 2149 | 1999 | 2137 | 1592 |
| Iceland | Other Europe | IS | | 20 | 10.4 | | 9.8 | | 14.3 | | 20.4 | | 20 | 19.8 | 18 | 21.4 | 215 | 190 | 209 | 156 |
| India |  | IN | | 33.5 | 27.8 | | 27.6 | | 22.8 | | 32.6 | | 31.9 | 30 | 26.1 | 34.1 | 2734 | 2622 | 2401 | 2139 |
| Indonesia* |  | ID | | 25 | 19.9 | |  | |  | |  | | 19.9 |  |  |  | 1 |  |  |  |
| Iran* |  | IR | |  | 0.1 | |  | |  | |  | | 0.1 |  |  |  | 1 |  |  |  |
| Ireland | EU27 | IE | | 12.5 | 14.2 | | 13.6 | | 13.2 | | 16.4 | | 13 | 12.7 | 12.4 | 13.8 | 1587 | 1509 | 1101 | 864 |
| Israel* |  | IL | | 25.1 | 5.7 | | 5.7 | | 5.7 | | 5.8 | | 19.8 | 18.9 | 18.9 | 22.2 | 13 | 13 | 13 | 13 |
| Italy | EU27 | IT | | 31.4 | 30.3 | | 35.1 | | 24 | | 41.1 | | 39.1 | 36.3 | 34.1 | 41.6 | 13073 | 13126 | 13959 | 11766 |
| Jamaica* |  | JM | | 28.3 | 13.4 | | 17.5 | |  | | 18.2 | | 14.7 | 21.5 |  | 21.8 | 3 | 2 |  | 2 |
| Japan |  | JP | | 37.2 | 30.1 | | 36.4 | | 28.8 | | 39.6 | | 39 | 41.1 | 36.5 | 43.3 | 8818 | 8409 | 8914 | 7952 |
| Jersey* |  | JE | | 4 | 32.9 | | 32.9 | |  | |  | | 32.9 | 32.9 |  |  | 1 | 1 |  |  |
| Kazakhstan |  | KZ | | 20 | 19.2 | | 25.3 | | 16.6 | | 28.1 | | 22.4 | 21.6 | 20 | 24 | 64 | 63 | 66 | 60 |
| Kosovo* | Other Europe | KV | |  | 17.7 | | 17.7 | | 17.3 | | 18.1 | | 11.3 | 11.4 | 10.5 | 12.5 | 6 | 6 | 6 | 6 |
| Latvia | EU27 | LV | | 15 | 10.2 | | 12.4 | | 9.3 | | 13.9 | | 15.8 | 15.4 | 15.1 | 16.4 | 1019 | 1001 | 1035 | 978 |
| Lebanon* |  | LB | | 15 | 16.1 | | 16 | | 15.8 | | 16.2 | | 16.1 | 16 | 15.8 | 16.2 | 1 | 1 | 1 | 1 |
| Liechtenstein* | Other Europe | LI | | 12.5 | 14.3 | | 13.6 | | 11.4 | | 17.9 | | 13.5 | 13.5 | 10.8 | 18 | 2 | 2 | 2 | 2 |
| Lithuania | EU27 | LT | | 15 | 11.6 | | 12.3 | | 10.1 | | 13.7 | | 15.1 | 14.4 | 13.4 | 15.9 | 729 | 715 | 490 | 488 |
| Luxembourg | EU27 | LU | | 29.1 | 1.7 | | 6.2 | | 1.2 | | 7.1 | | 10.8 | 19.5 | 4.9 | 26.8 | 1206 | 752 | 1172 | 627 |
| Madagascar* |  | MG | |  | 20.6 | |  | |  | |  | | 20.6 |  |  |  | 1 |  |  |  |
| Malaysia* |  | MY | | 24.8 | 17.6 | | 0.1 | | 0.1 | | 0.1 | | 10 | 0.1 | 0.1 | 0.1 | 5 | 1 | 1 | 1 |
| Malta | EU27 | MT | | 35 | 25.8 | | 25.3 | | 23 | | 26.1 | | 34.7 | 34.8 | 32.7 | 35 | 776 | 707 | 778 | 701 |
| Mauritius |  | MU | | 15 | 15 | | 10.5 | | 9.1 | | 19.2 | | 15.8 | 14.6 | 11.2 | 17.4 | 56 | 51 | 40 | 26 |
| Mexico |  | MX | | 30 | 25.4 | | 25 | | 22.9 | | 33.8 | | 28.8 | 27.3 | 23.2 | 31.9 | 251 | 213 | 209 | 160 |
| Moldova* | Other Europe | MD | | 12 | 5.1 | | 4.8 | |  | |  | | 13.8 | 10.1 |  |  | 15 | 11 |  |  |
| Monaco* | Other Europe | MC | |  | 29.5 | |  | |  | |  | | 29.5 |  |  |  | 1 |  |  |  |
| Montenegro | Other Europe | ME | | 9 | 10.6 | | 12.1 | | 9.6 | | 13.4 | | 9 | 8.5 | 8.4 | 9.2 | 77 | 76 | 77 | 72 |
| Morocco |  | MA | | 30.3 | 24.6 | | 23.2 | | 20.1 | | 28.7 | | 26.7 | 24.3 | 21.3 | 29.9 | 450 | 441 | 462 | 410 |
| Netherlands | EU27 | NL | | 25 | 10.4 | | 27.1 | | 7.9 | | 30.8 | | 23.7 | 23.8 | 19.7 | 25.2 | 3555 | 2759 | 3007 | 2066 |
| New Zealand |  | NZ | | 28 | 25.8 | | 25.2 | | 18.4 | | 30.4 | | 28.3 | 28.6 | 26.8 | 29.9 | 713 | 665 | 686 | 592 |
| North Macedonia | Other Europe | MK | | 10 | 5.5 | | 8.2 | | 4.3 | | 8.3 | | 8.2 | 8 | 6.7 | 9.7 | 161 | 153 | 163 | 143 |
| Norway | Other Europe | NO | | 27.6 | 50.3 | | 59.9 | | 43.9 | | 64.9 | | 27.7 | 27.4 | 24.3 | 29.5 | 6215 | 5301 | 6288 | 4905 |
| Pakistan* |  | PK | | 34.4 | 32.2 | | 28.3 | | 27.4 | | 31.3 | | 31.2 | 28.8 | 27.1 | 31.6 | 41 | 38 | 34 | 36 |
| Panama* |  | PA | | 25 | 30.3 | | 26.4 | | 25.9 | | 31.1 | | 29.8 | 26.4 | 25.6 | 30.8 | 4 | 4 | 4 | 4 |
| Paraguay* |  | PY | | 10 | 12.4 | | 14.7 | | 14.7 | | 14.8 | | 12.4 | 12.6 | 19.2 | 12.6 | 6 | 4 | 4 | 4 |
| Peru |  | PE | | 30 | 33 | | 31.5 | | 28.5 | | 37.1 | | 30.8 | 27.9 | 24.8 | 34.3 | 60 | 50 | 58 | 49 |
| Philippines |  | PH | | 30 | 21.3 | | 18.5 | | 18.5 | | 20.4 | | 30 | 28 | 28 | 29.6 | 729 | 751 | 752 | 739 |
| Poland | EU27 | PL | | 19 | 17 | | 17.9 | | 13.4 | | 20.8 | | 20.2 | 19.5 | 18.3 | 22 | 6051 | 5883 | 5477 | 5202 |
| Portugal | EU27 | PT | | 23.8 | 19.5 | | 17.5 | | 16 | | 21 | | 25 | 22.7 | 22.3 | 25.3 | 3839 | 3879 | 3920 | 3754 |
| Romania | EU27 | RO | | 16 | 17.5 | | 16.9 | | 13.3 | | 21.8 | | 17.2 | 16.1 | 13.4 | 20.6 | 3133 | 3106 | 3214 | 2483 |
| Russia |  | RU | | 20 | 17.8 | | 19.3 | | 16.3 | | 21.2 | | 19.3 | 19.1 | 18.2 | 20 | 6408 | 6055 | 6351 | 5930 |
| Rwanda* |  | RW | | 30 | 27.9 | | 25.7 | | 22.4 | | 33.2 | | 27.9 | 25.7 | 22.4 | 33.2 | 1 | 1 | 1 | 1 |
| Singapore |  | SG | | 17 | 6.2 | |  | |  | |  | | 12.2 |  |  |  | 826 |  |  |  |
| Slovakia | EU27 | SK | | 21 | 19.9 | | 21.4 | | 19 | | 24.2 | | 22.9 | 21.7 | 21.5 | 23.3 | 3242 | 3202 | 3232 | 3067 |
| Slovenia | EU27 | SI | | 17.8 | 14.4 | | 13.5 | | 11.5 | | 15.6 | | 17 | 16 | 14.3 | 18 | 1328 | 1272 | 1227 | 1102 |
| South Africa* |  | ZA | | 30.6 | 16 | |  | |  | |  | | 16 |  |  |  | 1 |  |  |  |
| South Korea |  | KR | | 23.8 | 20.6 | | 22.4 | | 15 | | 29 | | 21.6 | 22.3 | 16.4 | 26.8 | 1990 | 1878 | 2006 | 1511 |
| Spain | EU27 | ES | | 29.6 | 20.5 | | 22.3 | | 16.6 | | 26.3 | | 28 | 25.7 | 23.5 | 29.8 | 10537 | 9906 | 10668 | 9362 |
| Sri Lanka* |  | LK | | 28 | 28 | | 28 | | 24.7 | | 32.2 | | 12 | 20.1 | 11.2 | 21 | 25 | 23 | 24 | 21 |
| Sudan* |  | SD | | 35 | 8.3 | |  | |  | |  | | 8.3 |  |  |  | 1 |  |  |  |
| Sweden | EU27 | SE | | 23.7 | 13 | | 18.1 | | 10.8 | | 20.4 | | 20.7 | 20.3 | 18.1 | 22.4 | 9945 | 8829 | 8859 | 7679 |
| Switzerland* | Other Europe | CH | | 18 | 14.6 | | 6.4 | | 5.6 | | 9.4 | | 21.6 | 15 | 14.5 | 23.7 | 39 | 33 | 35 | 30 |
| Syria* |  | SY | | 24.4 | 83.5 | | 87.4 | | 81.1 | | 91.8 | | 83.5 | 87.4 | 81.1 | 91.8 | 1 | 1 | 1 | 1 |
| Taiwan* |  | TW | | 17 | 19.3 | | 18.8 | | 16.6 | | 22.8 | | 18.7 | 20.9 | 17.6 | 23.9 | 47 | 43 | 47 | 42 |
| Tanzania* |  | TZ | | 30 | 31.1 | | 31.3 | | 30.3 | | 32.1 | | 31.1 | 31.3 | 30.3 | 32.1 | 1 | 1 | 1 | 1 |
| Thailand |  | TH | | 22.6 | 17.2 | | 10.6 | | 6.1 | | 14.4 | | 19.8 | 19 | 19.2 | 20.1 | 203 | 200 | 118 | 196 |
| Trinidad and Tobago* |  | TT | | 25 | 26.1 | | 25.7 | | 25.7 | | 26.8 | | 26.3 | 25.8 | 25 | 27.1 | 3 | 3 | 3 | 3 |
| Tunisia* |  | TN | | 28 | 14.3 | | 7.4 | | 7.3 | | 14.9 | | 14.4 | 7.4 | 7.2 | 14.9 | 2 | 2 | 2 | 2 |
| Turkey |  | TR | | 20 | 18.6 | | 16.6 | | 9.4 | | 24 | | 20.3 | 16 | 9 | 27.8 | 454 | 442 | 461 | 250 |
| Ukraine | Other Europe | UA | | 20.2 | 21.6 | | 19.3 | | 18.6 | | 22.2 | | 21.4 | 20.7 | 20.4 | 21.9 | 783 | 771 | 782 | 761 |
| United Arab Emirates* |  | AE | | 55 | 96 | | 94.7 | | 94.7 | | 96.1 | | 96 | 94.7 | 94.7 | 96.1 | 1 | 1 | 1 | 1 |
| United Kingdom | Other Europe | GB | | 22.8 | 11.8 | | 19.7 | | 10.4 | | 21.3 | | 21.4 | 21.6 | 19.5 | 23.2 | 16619 | 14537 | 12818 | 8567 |
| United States* |  | US | | 40 | 5.8 | | 5.2 | | 17.8 | | 3.9 | | 32.5 | 24.1 | 23.1 | 29.1 | 39 | 36 | 25 | 24 |
| Uruguay |  | UY | | 25 | 9.7 | | 10.7 | | 8.7 | | 9.3 | | 20.1 | 21.4 | 15.5 | 23.6 | 98 | 98 | 95 | 84 |
| Uzbekistan* |  | UZ | |  | 15.2 | | 23.7 | | 9.6 | |  | | 15.2 | 23.7 | 9.6 |  | 1 | 1 | 1 |  |
| Vietnam* |  | VN | | 23.8 | 22.6 | | 22.6 | | 18.1 | | 28.9 | | 23.7 | 25.6 | 18 | 33 | 3 | 2 | 2 | 2 |
| Serbia | Other Europe | RS | | 13 | 8.1 | | 7.5 | | 6 | | 10.5 | | 11.8 | 10.5 | 8.2 | 14 | 885 | 864 | 903 | 741 |
| Zambia* |  | ZM | | 35 | 33.7 | | 25.6 | | 24.6 | | 35.5 | | 34.1 | 28.3 | 28.1 | 35.3 | 3 | 3 | 3 | 3 |
| Zimbabwe* |  | ZW | | 25.7 | 26.7 | | 26.8 | | 26.7 | | 26.9 | | 26.7 | 26.8 | 26.7 | 26.9 | 1 | 1 | 1 | 1 |

*Notes: Corporate income statutory tax rates (CIT), means and medians of ETRs in four estimations (ETR1–ETR4, defined in text) for 2011–2015. EU27 indicates whether a country was in the EU in February 2020 while Europe indicates non-EU countries geographically located mostly in Europe (i.e., this designation excludes Russia and Turkey). Countries marked with an asterisk (*) have fewer than 50 companies per sample. Source: Authors.*

**Table A2. Effective tax rates, robustness check (balanced sample)**

| Country name | EU | ISO2 | CIT (%) | | Mean (%) | | | | | | Median (%) | | | | Number of companies | | | |
| --- | --- | --- | --- | --- | --- | --- | --- | --- | --- | --- | --- | --- | --- | --- | --- | --- | --- | --- |
|  |  |  |  |  | **ETR1** | **ETR2** | **ETR3** | | **ETR4** | | **ETR1** | **ETR2** | **ETR3** | **ETR4** | **ETR1** | **ETR2** | **ETR3** | **ETR4** |
| Algeria* |  | DZ | | 25 | 11.7 | 12 | | 11 | | 13.1 | 22.9 | 22.3 | 20.3 | 24.9 | 30 | 30 | 30 | 30 |
| Argentina* |  | AR | | 35 | 38.2 | 37.5 | | 32 | | 48.4 | 35.4 | 35.2 | 33.6 | 40.3 | 16 | 16 | 16 | 16 |
| Australia |  | AU | | 30 | 30.9 | 28.6 | | 27.7 | | 32.8 | 29.7 | 27.8 | 26.5 | 30.9 | 963 | 963 | 963 | 963 |
| Austria | EU27 | AT | | 25 | 15.1 | 16.5 | | 13.9 | | 18.3 | 21.7 | 22.1 | 19.1 | 25 | 1493 | 1493 | 1493 | 1493 |
| Belgium | EU27 | BE | | 34 | 19.6 | 21.5 | | 15.8 | | 29.2 | 30.2 | 29.4 | 25.5 | 34.6 | 4656 | 4656 | 4656 | 4656 |
| Bermuda* |  | BM | | 0 | 18.6 | 36.8 | | 16.8 | | 46.5 | 18.6 | 36.8 | 16.8 | 46.5 | 1 | 1 | 1 | 1 |
| Bosnia and Herzegovina | Other Europe | BA | | 10 | 7 | 7.1 | | 6.5 | | 7.7 | 9.5 | 9.1 | 8.7 | 10.1 | 252 | 252 | 252 | 252 |
| Brazil |  | BR | | 34 | 23.8 | 21.6 | | 18.4 | | 29.5 | 27.7 | 22.9 | 19.5 | 33.4 | 307 | 307 | 307 | 307 |
| Bulgaria | EU27 | BG | | 10 | 9.8 | 8.7 | | 8 | | 10.9 | 10.1 | 9.5 | 9 | 10.6 | 717 | 717 | 717 | 717 |
| Burkina Faso* |  | BF | | 16 | 32.9 | 30 | | 29.7 | | 33.3 | 32.9 | 30 | 29.7 | 33.3 | 1 | 1 | 1 | 1 |
| Cape Verde* |  | CV | | 25 | 24.5 | 22.1 | | 21.6 | | 25.2 | 24.5 | 22.1 | 21.6 | 25.2 | 1 | 1 | 1 | 1 |
| Chile* |  | CL | | 20.5 | 20.2 | 19.8 | | 17.2 | | 23.9 | 19.4 | 17.1 | 14.5 | 22.2 | 18 | 18 | 18 | 18 |
| China |  | CN | | 25 | 19.5 | 19.6 | | 17.9 | | 21.9 | 21.2 | 20.2 | 19.6 | 22.6 | 3783 | 3783 | 3783 | 3783 |
| Colombia |  | CO | | 28.2 | 30.7 | 29.9 | | 25.1 | | 38.5 | 33.5 | 32.9 | 28.7 | 40.7 | 130 | 130 | 130 | 130 |
| Cote d'Ivoire* |  | CI | |  | 25.8 | 25 | | 25 | | 25.8 | 25.8 | 25 | 25 | 25.8 | 1 | 1 | 1 | 1 |
| Croatia | EU27 | HR | | 20 | 15 | 13.9 | | 11.9 | | 18.2 | 20.2 | 19.4 | 17.3 | 22 | 841 | 841 | 841 | 841 |
| Cyprus* | EU27 | CY | | 11.5 | 6.1 | 6.6 | | 5.5 | | 7.4 | 11.7 | 9.2 | 8.3 | 12.4 | 31 | 31 | 31 | 31 |
| Czechia | EU27 | CZ | | 19 | 16.1 | 17 | | 14.1 | | 20.1 | 19.4 | 18.4 | 16.3 | 22 | 3678 | 3678 | 3678 | 3678 |
| Denmark | EU27 | DK | | 24.3 | 18 | 28.8 | | 16.2 | | 33.5 | 24.7 | 24.2 | 21.9 | 26.1 | 2944 | 2944 | 2944 | 2944 |
| Ecuador* |  | EC | | 22.6 | 23.3 | 22.6 | | 21.9 | | 25.2 | 25.1 | 21.9 | 20 | 25.6 | 8 | 8 | 8 | 8 |
| Egypt* |  | EG | | 23.5 | 58.1 | 58.1 | | 57.6 | | 58.4 | 58.1 | 58.1 | 57.6 | 58.4 | 1 | 1 | 1 | 1 |
| El Salvador* |  | SV | | 30 | 36.9 | 30.5 | | 27.3 | | 43.1 | 36.9 | 30.5 | 27.3 | 43.1 | 1 | 1 | 1 | 1 |
| Estonia* | EU27 | EE | |  |  |  | |  | |  |  |  |  |  |  |  |  |  |
| Finland | EU27 | FI | | 23 | 13.6 | 15.2 | | 12.3 | | 17.3 | 22.1 | 21.9 | 20.4 | 23.3 | 2091 | 2091 | 2091 | 2091 |
| France | EU27 | FR | | 33.3 | 25 | 29.7 | | 22.5 | | 34.2 | 28.3 | 28.2 | 25.9 | 30.9 | 11081 | 11081 | 11081 | 11081 |
| Gabon* |  | GA | |  | 32.4 | 28.3 | | 27.6 | | 32.6 | 32.4 | 28.3 | 27.6 | 32.6 | 1 | 1 | 1 | 1 |
| Germany | EU27 | DE | | 29.5 | 20.9 | 22.6 | | 17.8 | | 27.8 | 27.9 | 26.2 | 23.5 | 30.2 | 6488 | 6488 | 6488 | 6488 |
| Greece | EU27 | GR | | 24.2 | 30 | 27.6 | | 26.5 | | 31.5 | 28.5 | 25.7 | 25.3 | 29.2 | 488 | 488 | 488 | 488 |
| Guyana* |  | GY | |  | 43 | 55.7 | | 55.6 | | 55.7 | 43 | 55.7 | 55.6 | 55.7 | 1 | 1 | 1 | 1 |
| Hong Kong* |  | HK | | 16.5 | 18 | 23.4 | | 18 | | 23.4 | 18 | 23.4 | 18 | 23.4 | 1 | 1 | 1 | 1 |
| Hungary | EU27 | HU | | 19 | 12.2 | 12 | | 9.3 | | 17.4 | 9.7 | 9.1 | 7.2 | 12.3 | 1237 | 1237 | 1237 | 1237 |
| Iceland | Other Europe | IS | | 20 | 18.4 | 15.9 | | 14.6 | | 20.4 | 20 | 19.9 | 18.4 | 21.7 | 95 | 95 | 95 | 95 |
| India |  | IN | | 33.5 | 27.5 | 27.7 | | 23.5 | | 33 | 30.9 | 28.5 | 25.1 | 34.1 | 1088 | 1088 | 1088 | 1088 |
| Ireland | EU27 | IE | | 12.5 | 18.4 | 17 | | 15.8 | | 19.4 | 13.2 | 12.6 | 11.8 | 14.3 | 490 | 490 | 490 | 490 |
| Italy | EU27 | IT | | 31.4 | 36.5 | 34.7 | | 31.2 | | 41.4 | 40.2 | 36.4 | 34.8 | 42.6 | 8867 | 8867 | 8867 | 8867 |
| Japan |  | JP | | 37.2 | 30.6 | 36 | | 29.4 | | 39.2 | 38.9 | 41.1 | 36.7 | 43.4 | 6836 | 6836 | 6836 | 6836 |
| Kazakhstan |  | KZ | | 20 | 23.1 | 24.2 | | 20.3 | | 28.2 | 21.5 | 21.4 | 19.9 | 24.3 | 54 | 54 | 54 | 54 |
| Kosovo* | Other Europe | KV | |  | 10.7 | 12.9 | | 10.2 | | 13.9 | 12.6 | 13.8 | 12.2 | 14.5 | 2 | 2 | 2 | 2 |
| Latvia | EU27 | LV | | 15 | 9.5 | 12.2 | | 8.8 | | 13.8 | 15.7 | 15.2 | 15 | 16 | 784 | 784 | 784 | 784 |
| Lebanon* |  | LB | | 15 | 16.1 | 16 | | 15.8 | | 16.2 | 16.1 | 16 | 15.8 | 16.2 | 1 | 1 | 1 | 1 |
| Liechtenstein* | Other Europe | LI | | 12.5 | 12 | 13.2 | | 9.6 | | 18.2 | 12 | 13.2 | 9.6 | 18.2 | 1 | 1 | 1 | 1 |
| Lithuania | EU27 | LT | | 15 | 11.8 | 11.7 | | 10.5 | | 13.4 | 15.2 | 14.2 | 13.5 | 16 | 346 | 346 | 346 | 346 |
| Luxembourg | EU27 | LU | | 29.1 | 4.3 | 6.8 | | 3.9 | | 8.2 | 23.9 | 22.7 | 18.6 | 28.4 | 326 | 326 | 326 | 326 |
| Malta | EU27 | MT | | 35 | 25 | 24.4 | | 22.5 | | 25.1 | 34.7 | 34.7 | 33 | 35 | 414 | 414 | 414 | 414 |
| Mauritius* |  | MU | | 15 | 17.6 | 6.1 | | 5.9 | | 18.7 | 17.6 | 6.1 | 5.9 | 18.7 | 1 | 1 | 1 | 1 |
| Mexico |  | MX | | 30 | 29.9 | 26.3 | | 24 | | 32.2 | 25.6 | 20.5 | 18.8 | 28.1 | 62 | 62 | 62 | 62 |
| Montenegro* | Other Europe | ME | | 9 | 10.8 | 12.8 | | 9.9 | | 14.2 | 8.5 | 8.3 | 7.6 | 9.9 | 16 | 16 | 16 | 16 |
| Morocco* |  | MA | | 30.3 | 31.9 | 29.1 | | 26.9 | | 35 | 24.5 | 22.5 | 22.2 | 24.8 | 44 | 44 | 44 | 44 |
| Netherlands | EU27 | NL | | 25 | 25.6 | 29.8 | | 22.6 | | 34 | 24.2 | 24.2 | 22.4 | 25.3 | 1261 | 1261 | 1261 | 1261 |
| New Zealand |  | NZ | | 28 | 24.1 | 26.1 | | 21.2 | | 30.5 | 28.3 | 29 | 26.9 | 30.4 | 285 | 285 | 285 | 285 |
| North Macedonia | Other Europe | MK | | 10 | 5.7 | 7.5 | | 5.4 | | 8.1 | 7.6 | 7.3 | 6.5 | 8.6 | 120 | 120 | 120 | 120 |
| Norway | Other Europe | NO | | 27.6 | 62.5 | 62.6 | | 58.7 | | 67.1 | 27.8 | 27.5 | 25.2 | 29.7 | 3555 | 3555 | 3555 | 3555 |
| Pakistan* |  | PK | | 34.4 | 33.2 | 29.6 | | 29.1 | | 33.8 | 31.4 | 30.5 | 29.5 | 33.8 | 23 | 23 | 23 | 23 |
| Panama* |  | PA | | 25 | 30.3 | 26.4 | | 25.9 | | 31.1 | 29.8 | 26.4 | 25.6 | 30.8 | 4 | 4 | 4 | 4 |
| Paraguay* |  | PY | | 10 | 36 | 24.4 | | 23.7 | | 37.6 | 36 | 24.4 | 23.7 | 37.6 | 1 | 1 | 1 | 1 |
| Peru* |  | PE | | 30 | 33.4 | 30.7 | | 27.9 | | 37.6 | 31.1 | 28.6 | 26.6 | 34.6 | 32 | 32 | 32 | 32 |
| Philippines |  | PH | | 30 | 21.9 | 20.4 | | 20.4 | | 21.2 | 30.1 | 28.1 | 28.1 | 29.6 | 551 | 551 | 551 | 551 |
| Poland | EU27 | PL | | 19 | 16.3 | 17.3 | | 14.1 | | 20.5 | 20.3 | 19.7 | 18.4 | 22.3 | 3402 | 3402 | 3402 | 3402 |
| Portugal | EU27 | PT | | 23.8 | 19.8 | 18 | | 16.8 | | 21.4 | 25.2 | 22.8 | 22.5 | 25.6 | 2834 | 2834 | 2834 | 2834 |
| Romania | EU27 | RO | | 16 | 17.3 | 16.8 | | 14.1 | | 21.6 | 16.8 | 16 | 13.9 | 20.7 | 1935 | 1935 | 1935 | 1935 |
| Russia |  | RU | | 20 | 18 | 19.3 | | 16.6 | | 21.4 | 19.2 | 19 | 18.2 | 20 | 4123 | 4123 | 4123 | 4123 |
| Slovakia | EU27 | SK | | 21 | 22.2 | 21.9 | | 21.5 | | 24.5 | 22.8 | 21.3 | 21.1 | 23.1 | 2412 | 2412 | 2412 | 2412 |
| Slovenia | EU27 | SI | | 17.8 | 13.7 | 13.5 | | 11.8 | | 16.1 | 17.2 | 16.2 | 15.1 | 18.7 | 745 | 745 | 745 | 745 |
| South Korea |  | KR | | 23.8 | 20 | 22.1 | | 16.5 | | 29.2 | 21.5 | 22.5 | 17.7 | 27.5 | 1140 | 1140 | 1140 | 1140 |
| Spain | EU27 | ES | | 29.6 | 21.8 | 23.2 | | 19 | | 27.4 | 28.2 | 26.1 | 24 | 30 | 6579 | 6579 | 6579 | 6579 |
| Sri Lanka* |  | LK | | 28 | 30.7 | 31.9 | | 29.1 | | 33.9 | 11 | 20.2 | 10.1 | 22 | 14 | 14 | 14 | 14 |
| Sweden | EU27 | SE | | 23.7 | 16.1 | 18.4 | | 14.5 | | 20.8 | 20.9 | 20.5 | 18.9 | 22.8 | 4810 | 4810 | 4810 | 4810 |
| Switzerland* | Other Europe | CH | | 18 | 7.9 | 6.4 | | 6.2 | | 8.2 | 20.2 | 15.5 | 14.9 | 23.7 | 24 | 24 | 24 | 24 |
| Taiwan* |  | TW | | 17 | 20.7 | 18.7 | | 17.1 | | 23.9 | 18.4 | 19.7 | 17.8 | 21 | 11 | 11 | 11 | 11 |
| Thailand* |  | TH | | 22.6 | 18.6 | 16.6 | | 15.1 | | 23.8 | 19.8 | 20.5 | 19.8 | 21.2 | 9 | 9 | 9 | 9 |
| Trinidad and Tobago* |  | TT | | 25 | 26.4 | 25.9 | | 25.8 | | 27.2 | 27.8 | 26.7 | 25.4 | 29.7 | 2 | 2 | 2 | 2 |
| Tunisia* |  | TN | | 28 | 14.3 | 7.4 | | 7.3 | | 14.9 | 14.4 | 7.4 | 7.2 | 14.9 | 2 | 2 | 2 | 2 |
| Turkey |  | TR | | 20 | 16 | 14.9 | | 11.4 | | 23.5 | 20 | 17.4 | 13.5 | 29 | 152 | 152 | 152 | 152 |
| Ukraine | Other Europe | UA | | 20.2 | 22.1 | 19.6 | | 19.1 | | 22.8 | 21.8 | 20.9 | 20.7 | 22.2 | 641 | 641 | 641 | 641 |
| United Arab Emirates* |  | AE | | 55 | 96 | 94.7 | | 94.7 | | 96.1 | 96 | 94.7 | 94.7 | 96.1 | 1 | 1 | 1 | 1 |
| United Kingdom | Other Europe | GB | | 22.8 | 16.3 | 20.2 | | 14.6 | | 22.9 | 22.2 | 22.1 | 20.2 | 23.9 | 5314 | 5314 | 5314 | 5314 |
| United States* |  | US | | 40 | 9.3 | 8.9 | | 8.9 | | 10.2 | 9.3 | 8.9 | 8.9 | 10.2 | 1 | 1 | 1 | 1 |
| Uruguay* |  | UY | | 25 | 3.6 | 3.8 | | 3.5 | | 4 | 15.5 | 15.4 | 14.3 | 18 | 22 | 22 | 22 | 22 |
| Serbia | Other Europe | RS | | 13 | 7.8 | 7.3 | | 6 | | 10.3 | 10.4 | 9.6 | 7.8 | 13.4 | 583 | 583 | 583 | 583 |

*Notes: Corporate income statutory tax rates (CIT), means and medians of ETRs in four estimations (ETR1–ETR4, defined in text) for 2011–2015. EU27 indicates whether a country was in the EU in February 2020 while Europe indicates non-EU countries geographically located mostly in Europe (i.e. this designation excludes Russia and Turkey). Countries marked with an asterisk (*) have fewer than 50 companies per sample. Source: Authors.*

**Table A3. Effective tax rates, robustness check (not removing observation when a company had negative profits in the previous year and using a balanced sample)**

| Country name | EU | ISO2 | CIT (%) | | Mean (%) | | | | | | | Median (%) | | | | | | Number of companies | | | |
| --- | --- | --- | --- | --- | --- | --- | --- | --- | --- | --- | --- | --- | --- | --- | --- | --- | --- | --- | --- | --- | --- |
|  |  |  |  |  | **ETR1** | **ETR2** | | **ETR3** | | **ETR4** | | **ETR1** | | **ETR2** | | **ETR3** | **ETR4** | **ETR1** | **ETR2** | **ETR3** | **ETR4** |
| Albania* | Other Europe | AL | | 12 | 25.5 | | 23.6 | | 22.8 | | 26.5 | | 15.8 | | 15.7 | 15.5 | 16.1 | 29 | 29 | 29 | 29 |
| Algeria |  | DZ | | 25 | 21.7 | | 21.1 | | 20.2 | | 22.9 | | 24.4 | | 23.4 | 22.4 | 25.7 | 117 | 117 | 117 | 117 |
| Argentina* |  | AR | | 35 | 37.2 | | 36.2 | | 31.8 | | 44.8 | | 35 | | 33.9 | 32.6 | 37.9 | 32 | 32 | 32 | 32 |
| Australia |  | AU | | 30 | 29 | | 26.6 | | 25.7 | | 30.8 | | 30 | | 28.3 | 26.9 | 31 | 1554 | 1554 | 1554 | 1554 |
| Austria | EU27 | AT | | 25 | 13.9 | | 14.9 | | 12.6 | | 16.8 | | 21.5 | | 21.7 | 18.9 | 24.8 | 1998 | 1998 | 1998 | 1998 |
| Belgium | EU27 | BE | | 34 | 20.2 | | 21.8 | | 16.4 | | 29.2 | | 29.9 | | 29 | 24.9 | 34.2 | 6619 | 6619 | 6619 | 6619 |
| Bermuda* |  | BM | | 0 | 18.6 | | 36.8 | | 16.8 | | 46.5 | | 18.6 | | 36.8 | 16.8 | 46.5 | 1 | 1 | 1 | 1 |
| Bolivia* |  | BO | | 25 | 4 | | 3 | | 3 | | 4 | | 4 | | 3 | 3 | 4 | 1 | 1 | 1 | 1 |
| Bosnia and Herzegovina | Other Europe | BA | | 10 | 7.1 | | 7.2 | | 6.6 | | 7.8 | | 9.8 | | 9.4 | 8.9 | 10.3 | 285 | 285 | 285 | 285 |
| Brazil |  | BR | | 34 | 22.9 | | 21.6 | | 18.2 | | 28.5 | | 26.3 | | 21.5 | 18.4 | 32.5 | 497 | 497 | 497 | 497 |
| Bulgaria | EU27 | BG | | 10 | 9.9 | | 8.8 | | 8.1 | | 11 | | 10.1 | | 9.7 | 9.4 | 10.6 | 986 | 986 | 986 | 986 |
| Burkina Faso* |  | BF | | 16 | 32.9 | | 30 | | 29.7 | | 33.3 | | 32.9 | | 30 | 29.7 | 33.3 | 1 | 1 | 1 | 1 |
| Cape Verde* |  | CV | | 25 | 24.5 | | 22.1 | | 21.6 | | 25.2 | | 24.5 | | 22.1 | 21.6 | 25.2 | 1 | 1 | 1 | 1 |
| Chile* |  | CL | | 20.5 | 21.5 | | 18.8 | | 17.3 | | 23.9 | | 19.1 | | 17 | 13.6 | 22.1 | 31 | 31 | 31 | 31 |
| China |  | CN | | 25 | 19.6 | | 19.3 | | 17.8 | | 21.6 | | 22.1 | | 20.6 | 19.9 | 23.6 | 6947 | 6947 | 6947 | 6947 |
| Colombia |  | CO | | 28.2 | 30.2 | | 29 | | 24.5 | | 37.8 | | 34.1 | | 32.5 | 28.7 | 41.3 | 169 | 169 | 169 | 169 |
| Cote d'Ivoire* |  | CI | |  | 20.6 | | 21.2 | | 18.7 | | 23.6 | | 20.8 | | 25 | 20.7 | 25.8 | 3 | 3 | 3 | 3 |
| Croatia | EU27 | HR | | 20 | 15.6 | | 14.8 | | 13.2 | | 17.9 | | 20.3 | | 19.7 | 17.8 | 22 | 1019 | 1019 | 1019 | 1019 |
| Cyprus | EU27 | CY | | 11.5 | 19.7 | | 11 | | 10.4 | | 13.3 | | 12.3 | | 10.3 | 9.8 | 12.7 | 84 | 84 | 84 | 84 |
| Czechia | EU27 | CZ | | 19 | 16.2 | | 16.9 | | 14.2 | | 20 | | 19.4 | | 18.5 | 16.5 | 21.9 | 4734 | 4734 | 4734 | 4734 |
| Denmark | EU27 | DK | | 24.3 | 17.3 | | 27.2 | | 15.6 | | 31.6 | | 24.7 | | 24.1 | 22.1 | 26.1 | 3890 | 3890 | 3890 | 3890 |
| Ecuador* |  | EC | | 22.6 | 24.6 | | 23.6 | | 23 | | 26.2 | | 26.5 | | 22.7 | 21.2 | 26.8 | 12 | 12 | 12 | 12 |
| Egypt* |  | EG | | 23.5 | 57.6 | | 57.5 | | 57 | | 57.9 | | 34.5 | | 33.4 | 33.1 | 34.7 | 2 | 2 | 2 | 2 |
| El Salvador* |  | SV | | 30 | 36.9 | | 30.5 | | 27.3 | | 43.1 | | 36.9 | | 30.5 | 27.3 | 43.1 | 1 | 1 | 1 | 1 |
| Estonia* | EU27 | EE | |  |  | |  | |  | |  | |  | |  |  |  |  |  |  |  |
| Finland | EU27 | FI | | 23 | 13.6 | | 15.2 | | 12.1 | | 17.4 | | 21.7 | | 21.2 | 20 | 23.1 | 2812 | 2812 | 2812 | 2812 |
| France | EU27 | FR | | 33.3 | 24.9 | | 29.3 | | 22.4 | | 33.8 | | 28.6 | | 28.4 | 26.2 | 31.1 | 14821 | 14821 | 14821 | 14821 |
| Gabon* |  | GA | |  | 15.9 | | 14.9 | | 14.6 | | 15.8 | | 22.4 | | 18.5 | 18 | 21.4 | 2 | 2 | 2 | 2 |
| Germany | EU27 | DE | | 29.5 | 20 | | 21.5 | | 17 | | 26.6 | | 27.5 | | 25.2 | 22.5 | 29.7 | 9091 | 9091 | 9091 | 9091 |
| Greece | EU27 | GR | | 24.2 | 28.8 | | 25.9 | | 24.8 | | 30.3 | | 28.8 | | 25.9 | 25.5 | 29.6 | 617 | 617 | 617 | 617 |
| Guyana* |  | GY | |  | 43 | | 55.7 | | 55.6 | | 55.7 | | 43 | | 55.7 | 55.6 | 55.7 | 1 | 1 | 1 | 1 |
| Hong Kong* |  | HK | | 16.5 | 17.3 | | 22.2 | | 17.3 | | 22.3 | | 10.1 | | 12.8 | 10.1 | 12.9 | 2 | 2 | 2 | 2 |
| Hungary | EU27 | HU | | 19 | 10.3 | | 10.8 | | 8.1 | | 15.2 | | 9.5 | | 9 | 7.2 | 11.7 | 1577 | 1577 | 1577 | 1577 |
| Iceland | Other Europe | IS | | 20 | 18.9 | | 16.5 | | 15.3 | | 20.4 | | 20 | | 19.6 | 18.1 | 21.4 | 154 | 154 | 154 | 154 |
| India |  | IN | | 33.5 | 27.9 | | 27.9 | | 24.1 | | 32.7 | | 32 | | 30 | 27.1 | 34.1 | 2071 | 2071 | 2071 | 2071 |
| Ireland | EU27 | IE | | 12.5 | 16.4 | | 15.3 | | 14.3 | | 17.3 | | 13 | | 12.6 | 11.8 | 14 | 698 | 698 | 698 | 698 |
| Israel* |  | IL | | 25.1 | 5.7 | | 5.7 | | 5.7 | | 5.8 | | 19.8 | | 18.9 | 18.9 | 22.2 | 13 | 13 | 13 | 13 |
| Italy | EU27 | IT | | 31.4 | 37 | | 34.8 | | 31.8 | | 41.1 | | 39.4 | | 35.9 | 34.5 | 41.6 | 11765 | 11765 | 11765 | 11765 |
| Japan |  | JP | | 37.2 | 31.1 | | 36.3 | | 29.7 | | 39.6 | | 38.9 | | 40.9 | 36.8 | 43.3 | 7952 | 7952 | 7952 | 7952 |
| Kazakhstan |  | KZ | | 20 | 23 | | 24.1 | | 20.2 | | 28.1 | | 22.1 | | 21.6 | 20.1 | 24.2 | 59 | 59 | 59 | 59 |
| Kosovo* | Other Europe | KV | |  | 17.7 | | 17.7 | | 17.3 | | 18.1 | | 11.3 | | 11.4 | 10.5 | 12.5 | 6 | 6 | 6 | 6 |
| Latvia | EU27 | LV | | 15 | 10.1 | | 12.4 | | 9.2 | | 13.9 | | 15.8 | | 15.4 | 15.1 | 16.4 | 978 | 978 | 978 | 978 |
| Lebanon* |  | LB | | 15 | 16.1 | | 16 | | 15.8 | | 16.2 | | 16.1 | | 16 | 15.8 | 16.2 | 1 | 1 | 1 | 1 |
| Liechtenstein* | Other Europe | LI | | 12.5 | 14.3 | | 13.6 | | 11.4 | | 17.9 | | 13.5 | | 13.5 | 10.8 | 18 | 2 | 2 | 2 | 2 |
| Lithuania | EU27 | LT | | 15 | 12.7 | | 12.2 | | 11.3 | | 13.8 | | 15.2 | | 14.2 | 13.4 | 15.9 | 453 | 453 | 453 | 453 |
| Luxembourg | EU27 | LU | | 29.1 | 5.1 | | 7.7 | | 4.5 | | 9.3 | | 22.6 | | 20.7 | 16.7 | 27.1 | 587 | 587 | 587 | 587 |
| Malaysia* |  | MY | | 24.8 | 0.1 | | 0.1 | | 0.1 | | 0.1 | | 0.1 | | 0.1 | 0.1 | 0.1 | 1 | 1 | 1 | 1 |
| Malta | EU27 | MT | | 35 | 25.7 | | 25.3 | | 23.5 | | 26.1 | | 34.8 | | 34.8 | 33.1 | 35 | 698 | 698 | 698 | 698 |
| Mauritius* |  | MU | | 15 | 16.8 | | 10.3 | | 9.3 | | 20.5 | | 16.7 | | 12.4 | 11 | 19.2 | 19 | 19 | 19 | 19 |
| Mexico |  | MX | | 30 | 29.1 | | 27.1 | | 23.5 | | 33.8 | | 28.2 | | 26.4 | 23.2 | 31.9 | 160 | 160 | 160 | 160 |
| Montenegro | Other Europe | ME | | 9 | 10.6 | | 12 | | 9.7 | | 13.4 | | 9.1 | | 8.5 | 8.5 | 9.2 | 72 | 72 | 72 | 72 |
| Morocco |  | MA | | 30.3 | 24.9 | | 23 | | 20.5 | | 28.7 | | 26.3 | | 24 | 21.3 | 29.9 | 410 | 410 | 410 | 410 |
| Netherlands | EU27 | NL | | 25 | 22 | | 26.6 | | 19.5 | | 30.3 | | 24.2 | | 24 | 22.2 | 25.3 | 1769 | 1769 | 1769 | 1769 |
| New Zealand |  | NZ | | 28 | 25.2 | | 25.7 | | 21.9 | | 30.4 | | 28.2 | | 28.6 | 26.8 | 30 | 581 | 581 | 581 | 581 |
| North Macedonia | Other Europe | MK | | 10 | 5.8 | | 7.5 | | 5.5 | | 8.3 | | 8.3 | | 8 | 7 | 9.7 | 143 | 143 | 143 | 143 |
| Norway | Other Europe | NO | | 27.6 | 60.3 | | 60.6 | | 56.6 | | 64.9 | | 27.8 | | 27.3 | 25.4 | 29.5 | 4904 | 4904 | 4904 | 4904 |
| Pakistan* |  | PK | | 34.4 | 30.5 | | 28 | | 26.9 | | 31.8 | | 30.9 | | 29.8 | 26.2 | 31.7 | 31 | 31 | 31 | 31 |
| Panama* |  | PA | | 25 | 30.3 | | 26.4 | | 25.9 | | 31.1 | | 29.8 | | 26.4 | 25.6 | 30.8 | 4 | 4 | 4 | 4 |
| Paraguay* |  | PY | | 10 | 14.3 | | 14.7 | | 14.7 | | 14.8 | | 14.1 | | 14.6 | 14.6 | 14.6 | 3 | 3 | 3 | 3 |
| Peru* |  | PE | | 30 | 33.8 | | 31.5 | | 29.1 | | 37.1 | | 31.1 | | 28 | 26.6 | 34.3 | 49 | 49 | 49 | 49 |
| Philippines |  | PH | | 30 | 21 | | 18.2 | | 18.2 | | 20.3 | | 30 | | 27 | 27 | 29.5 | 726 | 726 | 726 | 726 |
| Poland | EU27 | PL | | 19 | 16.2 | | 17.1 | | 14.1 | | 20.3 | | 20.2 | | 19.5 | 18.3 | 22.1 | 4724 | 4724 | 4724 | 4724 |
| Portugal | EU27 | PT | | 23.8 | 19.6 | | 17.4 | | 16.5 | | 21 | | 25 | | 22.5 | 22.3 | 25.3 | 3751 | 3751 | 3751 | 3751 |
| Romania | EU27 | RO | | 16 | 17.4 | | 16.9 | | 14.1 | | 21.8 | | 16.8 | | 16.1 | 14.2 | 20.6 | 2483 | 2483 | 2483 | 2483 |
| Russia |  | RU | | 20 | 18 | | 19.2 | | 16.6 | | 21.2 | | 19.3 | | 19.1 | 18.3 | 20 | 5842 | 5842 | 5842 | 5842 |
| Rwanda* |  | RW | | 30 | 27.9 | | 25.7 | | 22.4 | | 33.2 | | 27.9 | | 25.7 | 22.4 | 33.2 | 1 | 1 | 1 | 1 |
| Slovakia | EU27 | SK | | 21 | 22.1 | | 21.6 | | 21.3 | | 24.2 | | 22.9 | | 21.6 | 21.5 | 23.3 | 3067 | 3067 | 3067 | 3067 |
| Slovenia | EU27 | SI | | 17.8 | 13.3 | | 13.1 | | 11.4 | | 15.6 | | 17 | | 15.8 | 14.6 | 18.1 | 1043 | 1043 | 1043 | 1043 |
| South Korea |  | KR | | 23.8 | 20.1 | | 22.2 | | 16.7 | | 29 | | 21.4 | | 22.2 | 17.6 | 26.8 | 1511 | 1511 | 1511 | 1511 |
| Spain | EU27 | ES | | 29.6 | 21.5 | | 22.3 | | 18.7 | | 26.3 | | 28 | | 25.6 | 24 | 29.8 | 9360 | 9360 | 9360 | 9360 |
| Sri Lanka* |  | LK | | 28 | 28.7 | | 28.2 | | 25.5 | | 32.2 | | 12 | | 20.1 | 11.5 | 21 | 21 | 21 | 21 | 21 |
| Sweden | EU27 | SE | | 23.7 | 15.7 | | 18.2 | | 14.2 | | 20.5 | | 20.9 | | 20.3 | 18.7 | 22.6 | 6867 | 6867 | 6867 | 6867 |
| Switzerland* | Other Europe | CH | | 18 | 8.7 | | 7.1 | | 6.7 | | 9.4 | | 20.2 | | 15.5 | 14.9 | 23.7 | 30 | 30 | 30 | 30 |
| Syria* |  | SY | | 24.4 | 83.5 | | 87.4 | | 81.1 | | 91.8 | | 83.5 | | 87.4 | 81.1 | 91.8 | 1 | 1 | 1 | 1 |
| Taiwan* |  | TW | | 17 | 19 | | 18.8 | | 16.3 | | 22.8 | | 18.7 | | 20.7 | 17.6 | 23.9 | 42 | 42 | 42 | 42 |
| Tanzania* |  | TZ | | 30 | 31.1 | | 31.3 | | 30.3 | | 32.1 | | 31.1 | | 31.3 | 30.3 | 32.1 | 1 | 1 | 1 | 1 |
| Thailand |  | TH | | 22.6 | 12.2 | | 6.2 | | 6 | | 13.7 | | 19.8 | | 20.2 | 19.2 | 20.5 | 114 | 114 | 114 | 114 |
| Trinidad and Tobago* |  | TT | | 25 | 26.1 | | 25.7 | | 25.7 | | 26.8 | | 26.3 | | 25.8 | 25 | 27.1 | 3 | 3 | 3 | 3 |
| Tunisia* |  | TN | | 28 | 14.3 | | 7.4 | | 7.3 | | 14.9 | | 14.4 | | 7.4 | 7.2 | 14.9 | 2 | 2 | 2 | 2 |
| Turkey |  | TR | | 20 | 17.1 | | 16.3 | | 12.7 | | 24 | | 20.1 | | 18 | 13.8 | 27.8 | 250 | 250 | 250 | 250 |
| Ukraine | Other Europe | UA | | 20.2 | 21.5 | | 19.2 | | 18.7 | | 22.2 | | 21.5 | | 20.7 | 20.4 | 21.9 | 757 | 757 | 757 | 757 |
| United Arab Emirates* |  | AE | | 55 | 96 | | 94.7 | | 94.7 | | 96.1 | | 96 | | 94.7 | 94.7 | 96.1 | 1 | 1 | 1 | 1 |
| United Kingdom | Other Europe | GB | | 22.8 | 15.5 | | 18.9 | | 13.8 | | 21.5 | | 21.8 | | 21.7 | 20 | 23.6 | 7014 | 7014 | 7014 | 7014 |
| United States* |  | US | | 40 | 19.6 | | 14.4 | | 14.1 | | 20.2 | | 29.7 | | 15.7 | 15.7 | 31.9 | 19 | 19 | 19 | 19 |
| Uruguay |  | UY | | 25 | 7.6 | | 8.2 | | 7.2 | | 8.7 | | 19.3 | | 18 | 15.1 | 22.2 | 80 | 80 | 80 | 80 |
| Vietnam* |  | VN | | 23.8 | 21.9 | | 22.6 | | 18.1 | | 28.9 | | 21.7 | | 25.6 | 18 | 33 | 2 | 2 | 2 | 2 |
| Yugoslavia | Other Europe | RS | | 13 | 8 | | 7.5 | | 6.1 | | 10.5 | | 11.2 | | 10.5 | 8.6 | 14 | 741 | 741 | 741 | 741 |

*Notes: Corporate income statutory tax rates (CIT), means and medians of ETRs in four estimations (ETR1–ETR4, defined in text) for 2011–2015. EU27 indicates whether a country was in the EU in February 2020 while Europe indicates non-EU countries geographically located mostly in Europe (i.e., this designation excludes Russia and Turkey). Countries marked with an asterisk (*) have fewer than 50 companies per sample. Source: Authors.*

**Table A4. Effective tax rates, robustness check (keeping affiliates with 3 or more observations)**

| Country name | EU | ISO2 | CIT (%) | | Mean (%) | | | | | | | Median (%) | | | | | | Number of companies | | | |
| --- | --- | --- | --- | --- | --- | --- | --- | --- | --- | --- | --- | --- | --- | --- | --- | --- | --- | --- | --- | --- | --- |
|  |  |  |  |  | **ETR1** | **ETR2** | | **ETR3** | | **ETR4** | | **ETR1** | | **ETR2** | | **ETR3** | **ETR4** | **ETR1** | **ETR2** | **ETR3** | **ETR4** |
| Albania* | Other Europe | AL | | 12 | 14.4 | |  | |  | |  | | 14.4 | |  |  |  | 1 |  |  |  |
| Algeria* |  | DZ | | 25 | 25 | | 19.4 | | 17.7 | | 13.1 | | 23.2 | | 21.6 | 19.9 | 24.9 | 33 | 31 | 32 | 30 |
| Argentina* |  | AR | | 35 | 38.1 | | 38 | | 32.5 | | 48.4 | | 35.8 | | 36.6 | 33.4 | 39.1 | 31 | 22 | 25 | 17 |
| Armenia* |  | AM | | 20 | 22 | |  | |  | |  | | 22 | |  |  |  | 1 |  |  |  |
| Australia |  | AU | | 30 | 30.6 | | 28.4 | | 27 | | 32.8 | | 29.8 | | 28.4 | 26.8 | 30.8 | 1349 | 1324 | 1212 | 1054 |
| Austria | EU27 | AT | | 25 | 12.7 | | 17.1 | | 11.1 | | 18.3 | | 21.2 | | 22.6 | 18 | 25 | 1771 | 1636 | 1771 | 1498 |
| Barbados* |  | BB | | 25 | 1.8 | |  | | 1.8 | |  | | 1.8 | |  | 1.8 |  | 1 |  | 1 |  |
| Belgium | EU27 | BE | | 34 | 13.3 | | 21.6 | | 6.8 | | 29.2 | | 28.7 | | 29.2 | 22.6 | 34.6 | 5670 | 5226 | 5704 | 4689 |
| Bermuda* |  | BM | | 0 | 18.6 | | 36.8 | | 16.8 | | 46.5 | | 18.6 | | 36.8 | 16.8 | 46.5 | 1 | 1 | 1 | 1 |
| Bolivia* |  | BO | | 25 | 37.5 | |  | |  | |  | | 37.5 | |  |  |  | 1 |  |  |  |
| Bosnia and Herzegovina | Other Europe | BA | | 10 | 7 | | 7.1 | | 6.4 | | 7.7 | | 9.5 | | 9.2 | 8.7 | 10.1 | 274 | 261 | 275 | 252 |
| Brazil |  | BR | | 34 | 24.1 | | 21.7 | | 17.8 | | 29.5 | | 28.9 | | 22.9 | 19.3 | 33.4 | 376 | 345 | 372 | 310 |
| Bulgaria | EU27 | BG | | 10 | 9.6 | | 8.6 | | 5.9 | | 10.9 | | 10.1 | | 9.5 | 8.8 | 10.6 | 790 | 750 | 793 | 717 |
| Burkina Faso* |  | BF | | 16 | 32.9 | | 30 | | 29.7 | | 33.3 | | 32.9 | | 30 | 29.7 | 33.3 | 1 | 1 | 1 | 1 |
| Cambodia* |  | KH | | 20 | 4.6 | |  | |  | |  | | 4.6 | |  |  |  | 1 |  |  |  |
| Canada* |  | CA | | 26.6 | 21.8 | |  | |  | |  | | 21.8 | |  |  |  | 1 |  |  |  |
| Cape Verde* |  | CV | | 25 | 24.5 | | 22.1 | | 21.6 | | 25.2 | | 24.5 | | 22.1 | 21.6 | 25.2 | 1 | 1 | 1 | 1 |
| Chile* |  | CL | | 20.5 | 19.3 | | 19.8 | | 14.3 | | 23.9 | | 18.6 | | 17 | 13.6 | 22.2 | 22 | 19 | 21 | 18 |
| China |  | CN | | 25 | 19 | | 19.6 | | 17.4 | | 21.9 | | 21.2 | | 20.3 | 19.6 | 22.6 | 3884 | 3817 | 3866 | 3784 |
| Colombia |  | CO | | 28.2 | 30.1 | | 29.9 | | 22.1 | | 38.2 | | 33.2 | | 33.1 | 27 | 37.5 | 597 | 598 | 202 | 174 |
| Costa Rica* |  | CR | | 30 | 37.5 | |  | |  | |  | | 37.5 | |  |  |  | 1 |  |  |  |
| Cote d'Ivoire* |  | CI | |  | 25.8 | | 25 | | 25 | | 25.8 | | 25.8 | | 25 | 25 | 25.8 | 1 | 1 | 1 | 1 |
| Croatia | EU27 | HR | | 20 | 14 | | 13.7 | | 10.4 | | 18.2 | | 20.3 | | 19.1 | 16.4 | 22 | 980 | 936 | 991 | 841 |
| Cyprus* | EU27 | CY | | 11.5 | 6.2 | | 6.7 | | 5.5 | | 7.5 | | 12 | | 9.5 | 8.3 | 12.4 | 43 | 42 | 38 | 38 |
| Czechia | EU27 | CZ | | 19 | 13.8 | | 17.1 | | 10.7 | | 20.1 | | 19.4 | | 18.4 | 15.5 | 22 | 4285 | 4188 | 4327 | 3678 |
| Denmark | EU27 | DK | | 24.3 | 15.8 | | 28.6 | | 12.9 | | 33.4 | | 24.3 | | 24.2 | 19.7 | 26.1 | 4017 | 3269 | 3988 | 3011 |
| Dominica* |  | DM | |  | 28.2 | | 22.4 | |  | | 28.2 | | 28.2 | | 22.4 |  | 28.2 | 1 | 1 |  | 1 |
| Ecuador* |  | EC | | 22.6 | 23.3 | | 22.6 | | 21.9 | | 25.2 | | 25.1 | | 21.9 | 20 | 25.6 | 8 | 8 | 8 | 8 |
| Egypt* |  | EG | | 23.5 | 58.1 | | 58.1 | | 57.6 | | 58.4 | | 58.1 | | 58.1 | 57.6 | 58.4 | 1 | 1 | 1 | 1 |
| El Salvador* |  | SV | | 30 | 40 | | 28.3 | | 22.3 | | 43.1 | | 39.8 | | 28.7 | 23.5 | 43.1 | 2 | 2 | 2 | 1 |
| Estonia | EU27 | EE | | 20.8 | 14.1 | | 14.6 | |  | |  | | 15.1 | | 15.3 |  |  | 551 | 541 |  |  |
| Finland | EU27 | FI | | 23 | 12.3 | | 16.3 | | 10.2 | | 17.3 | | 21.9 | | 21.9 | 20 | 23.3 | 2393 | 2284 | 2362 | 2144 |
| France | EU27 | FR | | 33.3 | 16.2 | | 31.1 | | 12.1 | | 34.2 | | 27.7 | | 28.4 | 24.6 | 30.9 | 12764 | 11747 | 12853 | 11081 |
| Gabon* |  | GA | |  | 32.4 | | 28.3 | | 27.6 | | 32.6 | | 32.4 | | 28.3 | 27.6 | 32.6 | 1 | 1 | 1 | 1 |
| Germany | EU27 | DE | | 29.5 | 19.6 | | 23.9 | | 14.9 | | 27.8 | | 27.3 | | 26.3 | 22.1 | 30.1 | 7728 | 7213 | 7685 | 6592 |
| Greece | EU27 | GR | | 24.2 | 28.8 | | 27.9 | | 25.1 | | 31.5 | | 28.4 | | 25.7 | 25.2 | 29.2 | 498 | 515 | 522 | 488 |
| Guyana* |  | GY | |  | 43 | | 55.7 | | 55.6 | | 55.7 | | 43 | | 55.7 | 55.6 | 55.7 | 1 | 1 | 1 | 1 |
| Hong Kong* |  | HK | | 16.5 | 18 | | 23.4 | | 18 | | 23.4 | | 18 | | 23.4 | 18 | 23.4 | 1 | 1 | 1 | 1 |
| Hungary | EU27 | HU | | 19 | 9.2 | | 11.6 | | 6.1 | | 17.4 | | 9.9 | | 9.4 | 6.8 | 12.2 | 1589 | 1511 | 1587 | 1244 |
| Iceland | Other Europe | IS | | 20 | 8.5 | | 8 | | 14.5 | | 20.4 | | 20 | | 20 | 18.3 | 21.7 | 113 | 107 | 112 | 95 |
| India |  | IN | | 33.5 | 27 | | 27.2 | | 21.9 | | 33 | | 31.1 | | 27.5 | 23.4 | 34.1 | 1297 | 1271 | 1253 | 1094 |
| Ireland | EU27 | IE | | 12.5 | 16.1 | | 15.4 | | 14.3 | | 19.1 | | 13.2 | | 12.8 | 12.4 | 14.2 | 926 | 889 | 710 | 544 |
| Italy | EU27 | IT | | 31.4 | 29.8 | | 35.1 | | 23.3 | | 41.4 | | 39.8 | | 36.7 | 34.4 | 42.6 | 9880 | 9878 | 10486 | 8868 |
| Jamaica* |  | JM | | 28.3 | 12.9 | | 29 | |  | | 29 | | 19.4 | | 29 |  | 29 | 2 | 1 |  | 1 |
| Japan |  | JP | | 37.2 | 29.7 | | 36.2 | | 28.5 | | 39.2 | | 39 | | 41.3 | 36.4 | 43.4 | 7542 | 7212 | 7608 | 6836 |
| Kazakhstan |  | KZ | | 20 | 19.1 | | 25.3 | | 16.3 | | 28.2 | | 21.7 | | 21.6 | 19.9 | 24.3 | 57 | 55 | 57 | 54 |
| Kosovo* | Other Europe | KV | |  | 10.7 | | 12.9 | | 10.2 | | 13.9 | | 12.6 | | 13.8 | 12.2 | 14.5 | 2 | 2 | 2 | 2 |
| Latvia | EU27 | LV | | 15 | 9.5 | | 12.2 | | 8.7 | | 13.8 | | 15.7 | | 15.2 | 15 | 16 | 809 | 800 | 820 | 784 |
| Lebanon* |  | LB | | 15 | 16.1 | | 16 | | 15.8 | | 16.2 | | 16.1 | | 16 | 15.8 | 16.2 | 1 | 1 | 1 | 1 |
| Liechtenstein* | Other Europe | LI | | 12.5 | 12 | | 13.2 | | 9.6 | | 18.2 | | 12 | | 13.2 | 9.6 | 18.2 | 1 | 1 | 1 | 1 |
| Lithuania | EU27 | LT | | 15 | 10.7 | | 11.7 | | 9.2 | | 13.3 | | 15.2 | | 14.5 | 13.4 | 15.9 | 465 | 455 | 366 | 359 |
| Luxembourg | EU27 | LU | | 29.1 | 2.2 | | 7.4 | | 1.6 | | 8.2 | | 11.3 | | 21.8 | 6.7 | 28.4 | 620 | 395 | 615 | 334 |
| Malaysia* |  | MY | | 24.8 | 10 | |  | |  | |  | | 10 | |  |  |  | 1 |  |  |  |
| Malta | EU27 | MT | | 35 | 25 | | 24.5 | | 21.6 | | 25.1 | | 34.6 | | 34.6 | 32.3 | 35 | 455 | 421 | 458 | 416 |
| Mauritius* |  | MU | | 15 | 13.5 | | 6.1 | | 5.8 | | 18.7 | | 15.8 | | 6.1 | 5.6 | 18.7 | 3 | 3 | 3 | 1 |
| Mexico |  | MX | | 30 | 29.2 | | 25.3 | | 22.5 | | 32.2 | | 26.7 | | 21.9 | 19.5 | 28.1 | 82 | 73 | 72 | 62 |
| Moldova* | Other Europe | MD | | 12 | 11.9 | | 11.5 | |  | |  | | 9.6 | | 10.1 |  |  | 8 | 7 |  |  |
| Montenegro* | Other Europe | ME | | 9 | 10.8 | | 12.8 | | 9.9 | | 14.2 | | 8.7 | | 8.3 | 7.2 | 9.9 | 17 | 17 | 17 | 16 |
| Morocco* |  | MA | | 30.3 | 31.3 | | 29.3 | | 26.2 | | 35 | | 24.9 | | 23.6 | 23.2 | 24.8 | 47 | 45 | 47 | 44 |
| Netherlands | EU27 | NL | | 25 | 12.3 | | 29.6 | | 9.2 | | 33.5 | | 23.6 | | 24 | 19.7 | 25.3 | 2205 | 1746 | 1985 | 1418 |
| New Zealand |  | NZ | | 28 | 25.9 | | 26 | | 17.2 | | 30.5 | | 28.4 | | 29.1 | 26.8 | 30.4 | 320 | 295 | 322 | 285 |
| North Macedonia | Other Europe | MK | | 10 | 5.3 | | 8.2 | | 4.2 | | 8.1 | | 7.5 | | 7.2 | 6.1 | 8.6 | 137 | 128 | 138 | 120 |
| Norway | Other Europe | NO | | 27.6 | 52.6 | | 62.1 | | 46.3 | | 67.1 | | 27.7 | | 27.6 | 23.8 | 29.7 | 4510 | 3834 | 4552 | 3555 |
| Pakistan* |  | PK | | 34.4 | 34.2 | | 30.3 | | 29.7 | | 33.7 | | 31.5 | | 30.4 | 29.6 | 32.2 | 27 | 26 | 24 | 25 |
| Panama* |  | PA | | 25 | 30.3 | | 26.4 | | 25.9 | | 31.1 | | 29.8 | | 26.4 | 25.6 | 30.8 | 4 | 4 | 4 | 4 |
| Paraguay* |  | PY | | 10 | 36 | | 24.4 | | 23.7 | | 37.6 | | 36 | | 24.4 | 23.7 | 37.6 | 1 | 1 | 1 | 1 |
| Peru* |  | PE | | 30 | 32.5 | | 30.7 | | 27.1 | | 37.6 | | 31 | | 27.7 | 24.7 | 34.6 | 37 | 33 | 35 | 32 |
| Philippines |  | PH | | 30 | 21.8 | | 20.4 | | 20.4 | | 21.2 | | 30.1 | | 28.5 | 28.4 | 29.7 | 552 | 566 | 567 | 558 |
| Poland | EU27 | PL | | 19 | 16.8 | | 17.9 | | 13.5 | | 20.7 | | 20.4 | | 19.7 | 18.3 | 22.3 | 3851 | 3805 | 3744 | 3521 |
| Portugal | EU27 | PT | | 23.8 | 19.6 | | 18 | | 16.3 | | 21.4 | | 25.3 | | 22.9 | 22.5 | 25.6 | 2890 | 2914 | 2941 | 2834 |
| Romania | EU27 | RO | | 16 | 17.4 | | 16.7 | | 13.3 | | 21.6 | | 17.2 | | 16 | 13 | 20.7 | 2462 | 2444 | 2506 | 1935 |
| Russia |  | RU | | 20 | 17.9 | | 19.5 | | 16.4 | | 21.4 | | 19.2 | | 19.1 | 18.2 | 19.9 | 4443 | 4245 | 4409 | 4174 |
| Singapore |  | SG | | 17 | 7.2 | |  | |  | |  | | 12.4 | |  |  |  | 552 |  |  |  |
| Slovakia | EU27 | SK | | 21 | 20.6 | | 21.1 | | 21.3 | | 24.5 | | 22.7 | | 21.3 | 21.1 | 23.1 | 2539 | 2503 | 2522 | 2412 |
| Slovenia | EU27 | SI | | 17.8 | 13.9 | | 13.7 | | 11.7 | | 16.1 | | 17.3 | | 16.4 | 14.7 | 18.6 | 870 | 842 | 837 | 768 |
| South Africa* |  | ZA | | 30.6 | 16 | |  | |  | |  | | 16 | |  |  |  | 1 |  |  |  |
| South Korea |  | KR | | 23.8 | 20.8 | | 22.5 | | 15 | | 29.2 | | 21.7 | | 22.6 | 16.8 | 27.5 | 1483 | 1416 | 1490 | 1140 |
| Spain | EU27 | ES | | 29.6 | 20.7 | | 23 | | 16.5 | | 27.4 | | 28.1 | | 26.1 | 23.5 | 30 | 7363 | 6942 | 7412 | 6580 |
| Sri Lanka* |  | LK | | 28 | 29.5 | | 31.5 | | 27.6 | | 33.9 | | 11 | | 20.1 | 9.4 | 22 | 16 | 15 | 17 | 14 |
| Sweden | EU27 | SE | | 23.7 | 13.3 | | 18.2 | | 11 | | 20.8 | | 20.7 | | 20.7 | 18.4 | 22.7 | 6041 | 5462 | 5813 | 5018 |
| Switzerland* | Other Europe | CH | | 18 | 9.4 | | 5.8 | | 5.1 | | 8.2 | | 20.9 | | 15.1 | 14.8 | 23.7 | 30 | 26 | 27 | 24 |
| Taiwan* |  | TW | | 17 | 22.1 | | 18.7 | | 18.6 | | 23.9 | | 18.4 | | 19.7 | 18 | 21 | 12 | 11 | 12 | 11 |
| Thailand* |  | TH | | 22.6 | 18 | | 16.9 | | 15.1 | | 23.3 | | 19.8 | | 20.2 | 19.8 | 20.6 | 12 | 12 | 9 | 12 |
| Trinidad and Tobago* |  | TT | | 25 | 26.4 | | 25.9 | | 25.8 | | 27.2 | | 27.8 | | 26.7 | 25.4 | 29.7 | 2 | 2 | 2 | 2 |
| Tunisia* |  | TN | | 28 | 14.3 | | 7.4 | | 7.3 | | 14.9 | | 14.4 | | 7.4 | 7.2 | 14.9 | 2 | 2 | 2 | 2 |
| Turkey |  | TR | | 20 | 16 | | 14 | | 7.1 | | 23.5 | | 20 | | 15.3 | 8.2 | 29 | 293 | 287 | 295 | 152 |
| Ukraine | Other Europe | UA | | 20.2 | 22 | | 19.7 | | 19.2 | | 22.8 | | 21.6 | | 20.9 | 20.7 | 22.2 | 651 | 647 | 657 | 641 |
| United Arab Emirates* |  | AE | | 55 | 96 | | 94.7 | | 94.7 | | 96.1 | | 96 | | 94.7 | 94.7 | 96.1 | 1 | 1 | 1 | 1 |
| United Kingdom | Other Europe | GB | | 22.8 | 13.7 | | 22.7 | | 12 | | 22.9 | | 22.1 | | 22.5 | 19.7 | 23.8 | 10033 | 8895 | 8595 | 5985 |
| United States* |  | US | | 40 | 9.3 | | 8.9 | | 8.9 | | 10.2 | | 9.3 | | 8.9 | 8.9 | 10.2 | 1 | 1 | 1 | 1 |
| Uruguay* |  | UY | | 25 | 3.6 | | 3.8 | | 3.5 | | 4 | | 17.6 | | 16.3 | 14.3 | 18 | 26 | 26 | 26 | 22 |
| Uzbekistan* |  | UZ | |  | 15.2 | | 23.7 | | 9.6 | |  | | 15.2 | | 23.7 | 9.6 |  | 1 | 1 | 1 |  |
| Serbia | Other Europe | RS | | 13 | 7.7 | | 7.3 | | 5.7 | | 10.3 | | 10.7 | | 9.6 | 7.4 | 13.4 | 680 | 671 | 691 | 583 |

*Notes: Corporate income statutory tax rates (CIT), means and medians of ETRs in four estimations (ETR1–ETR4, defined in text) for 2011–2015. EU27 indicates whether a country was in the EU in February 2020 while Europe indicates non-EU countries geographically located mostly in Europe (i.e., this designation excludes Russia and Turkey). Countries marked with an asterisk (*) have fewer than 50 companies per sample. Source: Authors.*

**Table A5. Country-specific details related to corporate income taxation**

| **Country** | **Country-specific details** |
| --- | --- |
| **Australia** | A 28.5% tax rate applies for years beginning on or after 1 July 2015 for business entities with aggregate turnover of less than AUD 2 million (approx. EUR 1.2 million). From 2012, a tax rate of 40% is imposed on profits from petroleum companies. Between 2012 and 2014, a rate of 40% was imposed on profits from mining companies (EY, 2016). |
| **Austria** | All companies, including those incurring tax losses, are subject to a minimum tax. The minimum tax is EUR 1,750 for an Austrian private limited company; EUR 3,500 for a stock corporation; EUR 6,000 for a European stock corporation and EUR 5,452 for banks and insurance companies (EY, 2016). |
| **Belgium** | A surtax of 3% is levied for crisis contribution. Progressive tax schedule from 24.25% to 34% for companies with income below EUR 322,500. Tax incentive for audio visual investments permits to deduct 310% of the investment from the tax base (EY, 2016). |
| **Bosnia and Herzegovina** | There are two main tax jurisdictions in Bosnia and Herzegovina (BiH): the Federation of BiH (FBiH) and the Republika Srpska (RS). The FBiH offers a 50% decrease in the corporate tax liability if a company invests more than BAM 20 million in production assets during a five-year period; a 30% decrease in the corporate tax liability if a company invests an amount exceeding 50% of the value of its pre-tax profits in equipment for production, and the equipment is financed from the company’s own assets; and a double tax deduction for salary expenses related to hiring of new employees for a period exceeding 12 months. The RS offers a decrease in the corporate tax liability if a company invests in equipment for production necessary for carrying out a registered business activity (Deloitte, 2021). |
| **Brazil** | Exemption from, or reduction of, CIT is granted to businesses in certain underdeveloped areas. A Social Contribution Tax is levied at a general rate of 9%. For financial institutions, private insurance companies and capitalization companies, the rate is 20% while for credit unions, the rate is 17% (EY, 2016). |
| **Bulgaria** | A 10% tax is imposed on certain expenses, such as employee-related, in-kind fringe benefits and representation-related expenses (EY, 2016). |
| **China** | A tax rate of 15% applies to new technology enterprises. A 10% tax rate applies to software enterprises, animation and comics enterprises and integrated circuit designing enterprises. A five-year tax holiday is granted to both groups of enterprises (EY, 2016). |
| **Colombia** | A tax rate of 15% applies to legal entities in free-trade zones. Reduced and gradually increasing tax rates apply to small businesses that begin operations on or after January 1, 2011. 0% applies for the first two years, an increase by 25% from the regular tax rate applies from the third to the fifth year, and the regular corporate income tax rate applies from the sixth year. The income tax for equality rate is 9% (EY, 2016). |
| **Croatia** | Tax exemptions and other tax reliefs are available for investments in new business activities and new workplaces (EY, 2016). |
| **Cyprus** | There are no special features applicable to the tax rate or the tax base other than the standard corporate tax. |
| **Czechia** | Basic investment funds are subject to a 5% tax rate and pension funds, to a 0% tax rate. Investment incentives (e.g. corporate income tax relief for 10 years and real estate tax exemption) are available to investors launching or expanding manufacturing production, technology centers and business support services centers (EY, 2016). |
| **Denmark** | There are no special features applicable to the tax rate or the tax base other than the standard corporate tax. |
| **Estonia** | Undistributed profit in Estonia is not taxed. |
| **Finland** | There are no special features applicable to the tax rate or the tax base other than the standard corporate tax. |
| **France** | The taxation of French companies is based on a territorial principle. As a result, French companies carrying on a business outside France are generally not taxed in France on the related profits. However, under the French CFC rules, income earned by a French enterprise through a foreign enterprise may be taxed in France if such income is subject to an effective tax rate that is 50% lower than the French effective tax rate on similar income. A social security surtax of 3.3% is imposed on the corporate income tax amount. Between 2013 and 2016, a temporary surtax of 10.7% was imposed on the corporate income tax amount for companies with turnover exceeding EUR 250 million (EY, 2016). |
| **Germany** | About half of the 29.5% CIT rate is obtained as an average rate computed across selected municipalities of the Gewerbesteuer tax rate, which does not allow 100% deduction of financial costs. Research interest has recently surged, in part in response to this paper’s preliminary results used in a policy report [57] which sheds further light on why the estimated ETR range is rather wide (see e.g. in German literature, Bräutigam, Ludwig, & Spengel, 2019, and Huber & Maiterth 2019). |
| **Greece** | There are no special features applicable to the tax rate or the tax base other than the standard corporate tax. |
| **Hungary** | A 10% tax rate applies to the first HUF 500 million (approx. USD 1,720,000) of taxable income. An alternative minimum tax regime is in force. Tax incentives such as R&D double and triple deduction and tax allowance for development projects. Tax relief is provided to corporate taxpayers supporting sport or cultural organizations and sponsoring film production (EY, 2016). |
| **Iceland** | The rate for taxable partnerships is 36% (EY, 2016). |
| **India** | If a company (domestic or foreign) exceeds INR10 million of net income, a surcharge between 2% and 12% will be applied. A cess of 3% is imposed. Tax incentives apply (e.g. accelerated deduction of capital expenditure; a 10-year tax holiday for enterprises engaged in infrastructure facilities, in developing a Special Economic Zone, in processing, preserving and packaging of fruits or vegetables and from the integrated business of handling, storing and transporting food grains; a 15-year tax holiday for enterprises engaged in export activities that begins to manufacture, produce articles or provide services in Special Economic Zones; and a 5-year tax holiday for business engaged in collecting, processing or treating biodegradable waste (EY, 2016). |
| **Ireland** | The ETRs higher than statutory rates are consistent with existing literature; for example, the Irish public audit body has recently found ETRs of similar magnitude (Comptroller and Auditor General, 2017, in particular pp 294, 298 and 299). Orbis data does not account for the case of the Apple company [21]. The audit body also counters the evidence of low taxation in Ireland presented by e.g. Stewart (2014). |
| **Italy** | The statutory rate for Italy (31.4%) includes both the standard corporate tax (IRES) and the regional business tax (IRAP). The tax base for IRAP, though, differs from IRES (because IRAP does not allow deducting personnel costs, interests on leasing, and some devaluations). (That 31.4% value is based on a number of assumptions in order to transform the IRAP liability into an equivalent IRES tax rate.) |
| **Japan** | Local income taxes are also imposed (EY, 2016). |
| **Kazakhstan** | Permanent establishments are subject to a 15% branch profits tax on their profits after deduction of corporate income tax. Businesses engaging in the exploration and extraction of mineral resources operate under a particular tax regime. Expenditure on certain fixed assets can be deducted in the first three years after commissioning. Alternatively, it can be deducted in full in the tax year in which the expenditure is incurred. A reduction of the corporate income tax payable by 100% is applied to entities carrying out their activities in special-economic zones (EY, 2016). |
| **Latvia** | Companies that operate in special-economic zones benefit from an 80% rebate of corporate income tax on income derived from their operations in these zones. Companies that invest more than EUR 10 million in long-term investment projects may apply for corporate income tax rebates of 25% and 50% of the initial investment amount (EY, 2016). |
| **Lithuania** | Reduced rates apply to small (5%), agricultural (5%) and social (0%) companies and to companies registered and operating in free-economic zones (0%) that satisfy certain conditions (EY, 2016). |
| **Luxembourg** | Corporate income tax rates vary depending on the income level. A municipal business tax and an additional employment fund contribution are levied on income. All corporate entities having their statutory seat or central administration in Luxembourg are subject to a minimum tax of EUR 3,000 (EY, 2016). |
| **Malta** | Companies engaged in specified activities can benefit from tax credits regarding capital expenditure, job creation or reinvestment of profits derived from business in an approved project. The Income Tax Act provides for a deduction of 150% of research and development expenditure incurred (EY, 2016). In Malta operates a full imputation and tax refund system which grants a shareholder the right to claim a refund of all or a part of the corporate tax paid on the qualifying profits out of which dividends were distributed (Deloitte, 2021). |
| **Mexico** | The income tax law recognizes the effects of inflation on depreciation of fixed assets, cost on sales of fixed assets, sales of capital stock (shares), monetary assets and liabilities and tax loss carry forwards. Investment in capital stock may be adjusted for inflation at the time of capital stock reductions or liquidation. Taxes are also indexed for inflation in certain circumstances (EY, 2016). |
| **Morocco** | Corporate income tax is imposed at proportional rates ranging from 10% to 31%. The corporate income tax rate is 37% for banks, financial institutions, and insurance companies. Tax exemptions and tax reductions are available to agricultural enterprises, export companies, hotel and. mining companies. Moreover, companies holding a hydrocarbon exploration and exploitation permit are exempt from corporate income tax for 10 years from the beginning of production. Banks and holding companies located in offshore zones benefit from a reduction in corporate income tax for the first 15 years of operation. Banks may elect to pay a minimum corporate income tax of USD 25,000 or pay tax at a reduced rate of 10%. Holding companies pay a flat tax of USD 5,000 per year (EY, 2016). |
| **Netherlands** | A tax rate of 20% applies to the first EUR 200,000 of taxable income. The Innovation Box is available, allowing net income from certain intellectual property to be effectively taxed at a rate of 5%.. This rate applies only to the extent that the net earnings derived from the self developed intangible assets exceed the development costs (EY, 2016). |
| **New Zealand** | There are no special features applicable to the tax rate or the tax base other than the standard corporate tax. |
| **North Macedonia** | Companies are exempt from income tax for the first 10 years of their activities in a Technological Industrial Development Zone. As of January 2015, companies may claim tax relief for the amount of profits reinvested in business-related tangible and intangible assets. No relief is available for profits reinvested in cars, furniture, carpets, audio visual devices and other decorative objects used to equip administrative premises (EY, 2016). |
| **Norway** | Special tax provisions apply to the Norwegian petroleum sector, which is subject to a marginal tax rate of up to 78% (The Norwegian Tax Administration, 2018). |
| **Peru** | Mining companies are subject to an additional Special Mining Tax considering progressive marginal rates ranging from 2% to 8.4%. The tax is imposed on the operating profits derived from sales of metallic mineral resources. Mining companies that have signed stability agreements with the state are subject to voluntary payments considering progressive marginal rates ranging from 4% to 13.12%. These rates are applied to the operating profits derived from sales of metallic mineral resources. Tax incentives are available for investments in mining enterprises, oil and gas licences and services contracts and certain agricultural activities. They are also available for investments in manufacturing industries located in the jungle, in designated tax-free zones and in borderline areas of the country (EY, 2016). |
| **Philippines** | Subject to certain exceptions, a 2% Minimum Corporate Income Tax (MCIT) may be imposed on domestic and resident foreign corporations beginning with the fourth tax year following the year of commencement of business operations. The MCIT must be paid if the corporation has zero or negative taxable income or if the MCIT is greater than the regular corporate income tax liability. Philippine-source income of foreign corporations are taxed at preferential rates (EY, 2016). |
| **Poland** | Withholding tax is not imposed on transfers of profits from a branch to its head office (EY, 2016). |
| **Portugal** | Small and medium-sized companies can benefit from a 17% reduced rate for the first EUR 15,000 of taxable profit. A municipal surcharge of 1.5% is generally imposed on the taxable profit determined for corporate income tax purposes. A state surcharge of 3% is imposed on the taxable profit between EUR1,500,000 and EUR7,500,000. If the taxable profit exceeds EUR7,500,000, the state surcharge is levied at a rate of 5% on the excess up to EUR35 million. If the taxable profit exceeds EUR35 million, the state surcharge is levied at a rate of 7% on the excess (EY, 2016). |
| **Romania** | Income derived by companies from night bars, nightclubs, discos and casinos directly or in association may not be less than 5% of the gross income derived from their activities. Romania offers certain tax incentives such as accelerated depreciation, reinvested profit, allowance for research and development activities. Incentives are also available to titleholders of oil and gas concessions (EY, 2016). |
| **Russia** | For both Russian legal entities and foreign legal entities, the basic corporate profits tax rate consists of a 2% rate payable to the federal government and rates ranging from 13.5% to 18% payable to the regional governments. The regional governments  set the rates applicable to their respective regions. As a result, the basic corporate profits tax rate varies from 15.5% to  20%, depending on the rate set by the regional government. A 0% tax rate applies to profits of Russian companies performing educational activities and medical activities if they satisfy certain criteria (EY, 2016). |
| **Serbia** | A company qualifies for a 10-year tax exemption if it invests RSD 1 billion (approximately EUR 9 million) in its own fixed assets and if it employs at least 100 new workers in the period of investment (EY, 2016). |
| **Singapore** | Seventy-five percent of the first SGD 10,000 of normal chargeable income is exempt from tax, and 50% of the next SGD290,000 is exempt from tax. Various tax incentives, exemptions and reductions are available for R&D expenditure, equity investments, pioneer enterprises, headquarters, financial and maritime companies (EY, 2016). |
| **Slovakia** | Minimum tax regime is available. The Slovak Republic provides incentives such as tax reliefs and transfers of immovable assets owned by the state or municipality at a price lower than the market price (EY, 2016). |
| **Slovenia** | The corporate income tax rate for qualified venture capital companies is 0%, subject to specific conditions. Investment funds that distribute 90% of their operating profits for the preceding tax year by 30 November of the current tax year are taxed at a rate of 0%. Pension funds are taxed at a rate of 0% (EY, 2016). |
| **South Korea** | South Korea’s progressive tax schedule (OECD, 2018b). |
| **Spain** | Newly incorporated entities carrying out business activities are taxed at a rate of 15% in the first fiscal year in which the entity has a positive tax base and in the following year, regardless of the amount of the tax base. Companies licensed to operate in  the Canary Islands Special Zone are subject to a reduced tax rate of 4% if certain conditions are satisfied. Tax rate applicable to income from reinsurance operations is 1.5%. A 4% tax rate applies to Spanish-source income generated by companies resident abroad operating ships and aircraft in Spain. Interest income is exempt from tax if the recipient is resident in  an EU member state that is not on the Spanish tax haven list (EY, 2016). |
| **Sweden** | There are no special features applicable to the tax rate or the tax base other than the standard corporate tax. |
| **Turkey** | Incentive regulations provide for a wide range of incentive and support elements for certain investments with incentive certificates, including reduced corporate tax rates, government support for interest on loans, government support for employees’ and employers’ shares of social security premiums, government support for income tax for wages, value-added tax (VAT) and customs duty exemptions, VAT refund support and allocation of treasury-owned lots (EY, 2016). |
| **Ukraine** | In addition to the general CIT rate, insurance companies also pay special CIT of 0% and 3% on their income. Long-term life insurance premiums, insurance premiums under voluntary pension programmes, and voluntary medical insurance premiums are subject to the 0% rate; the 3% rate applies to all other insurance premiums received by the insurance company (PwC, 2021). |
| **United Kingdom** | Small profits rate of 19% for ring-fence profits applies in certain circumstances if taxable profits are below GBP300,000. This benefit is phased out for taxable profits from GBP300,000 to GBP1,500,000. These limits are reduced if associated companies exist. A special rate of corporation tax of 45% applies on restitution interest, which is compound interest received from the UK tax authorities on the repayment of tax (EY, 2016). |

*Notes:* *Country-specific details related to corporate income taxation listed in the table have been reviewed based on a combination of existing information sources, academic literature, international organisations as well as big accounting companies.* The table shows a *brief overview of corporate tax system characteristics that are more likely to be consequential for the estimated ETRs and their differences with statutory rates. For each country, the table lists the characteristics of the corporate income tax systems that differ across various parts of the country (e.g.,* *municipalities in Germany), for different types of companies (e.g. progressive taxation in South Korea) or other atypical features that define the tax rate or the taxable base differently from the standard corporate tax. We provide these characteristics for countries that have more than 50 companies per sample (i.e., not marked with an asterisk (*) in the results tables) and therefore with more reliable estimates of ETRs. For the sake of length and clarity, the table is not supposed to be comprehensive but should highlight the most important examples, if any, for each country. These country-specific examples should make the reader aware of the intrinsic limitations of our proposed methodology, as well as any other cross-country empirical work analysing taxation using the Orbis data. Source: Authors.*

**Table A6. Statistical Classification of Economic Activities in the European Community Rev. 2, Level 1 Codes**

| **NACE code** | **Economic area** |
| --- | --- |
| A | Agriculture, Forestry and Fishing |
| B | Mining and Quarrying |
| C | Manufacturing |
| D | Electricity, Gas, Steam and Air Conditioning Supply |
| E | Water Supply; Sewerage, Waste Management and Remediation Activities |
| F | Construction |
| G | Wholesale and Retail Trade; Repair of Motor Vehicles and Motorcycles |
| H | Transportation and Storage |
| I | Accommodation and Food Service Activities |
| J | Information and Communication |
| K | Financial and Insurance Activities |
| L | Real Estate Activities |
| M | Professional, Scientific and Technical Activities |
| N | Administrative and Support Service Activities |
| O | Public Administration and Defence; Compulsory Social Security |
| P | Education |
| Q | Human Health and Social Work Activities |
| R | Arts, Entertainment and Recreation |
| S | Other Service Activities |
| T | Activities of Households as Employers; Undifferentiated Goods and Services Producing Activities of Households for Own Use |
| U | Activities of Extraterritorial Organisations and Bodies |

*Source: [69]*

**References - Appendix**

*69.* Eurostat. NACE Rev. 2, Statistical classification of economic activities in the European Community. Gen Reg Stat. 2008. Available: https://ec.europa.eu/eurostat/documents/3859598/5902521/KS-RA-07-015- EN.PDF.pdf/dd5443f5-b886-40e4-920d-9df03590ff91?t=1414781457000
